# FBXO44 Regulates FOXP1 Degradation Through AURKA‐Dependent Phosphorylation to Promote Colorectal Cancer Progression

**DOI:** 10.1002/advs.202415458

**Published:** 2025-10-06

**Authors:** Hongxu Nie, Hengjie Xu, Sheng Yang, Chuanxin Tian, Tuo Wang, Chi Jin, Zhihao Chen, Xiaowei Wang, Junwei Tang, Yifei Feng, Yueming Sun

**Affiliations:** ^1^ Department of General Surgery Colorectal Institute of Nanjing Medical University The First Affiliated Hospital of Nanjing Medical University Nanjing 210029 China; ^2^ Jiangsu Province Engineering Research Center of Colorectal Cancer Precision Medicine and Translational Medicine Nanjing 210029 China; ^3^ Collaborative Innovation Center for Cancer Personalized Medicine Nanjing Medical University Nanjing 210029 China; ^4^ Department of General Surgery The Third Affiliated Hospital of Nanjing Medical University Changzhou 213100 China; ^5^ Department of General Surgery, Qilu Hospital Shandong University Jinan 250012 China

**Keywords:** colorectal cancer, FBXO44, FOXP1, phosphorylation, ubiquitination

## Abstract

Emerging evidence highlights the role of SCF E3 ligases, consisting of SKP1, cullin‐1, and F‐box proteins, in cancer biology by regulating the ubiquitination and degradation of key proteins. This study identifies F‐box only protein 44 (FBXO44) as an oncogene in colorectal cancer (CRC). FBXO44 is upregulated in CRC patients and correlates with poor prognosis. Knockdown of FBXO44 inhibits CRC cell proliferation and organoid growth, as well as xenograft tumor growth and AOM/DSS‐induced intestinal tumorigenesis. Conversely, FBXO44 overexpression accelerates tumor growth in vitro and in vivo. Mechanistically, FBXO44 targets Forkhead box protein P1 (FOXP1) for degradation. Aurora kinase A (AURKA) phosphorylates FOXP1 at Ser440, enhancing FBXO44 binding, leading to K48‐linked ubiquitination at K377 and proteasomal degradation. This degradation relieves FOXP1 repression of Cyclin E2, promoting CRC cell proliferation. In summary, FBXO44 is an oncogene that promotes CRC tumorigenesis by degrading FOXP1 and upregulating Cyclin E2, offering a potential therapeutic target for CRC.

## Graphical abstract

FBXO44 promotes colorectal cancer progression by targeting FOXP1 for ubiquitin‐mediated degradation. This study reveals a phosphorylation‐dependent mechanism involving AURKA and highlights the FBXO44/FOXP1/Cyclin E2 axis as a potential therapeutic target in colorectal cancer.

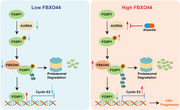

## 1 Introduction

Colorectal cancer (CRC) ranks among the most prevalent malignancies globally, ranking third in incidence among both men and women, and second in mortality. Notably, the incidence of CRC is rapidly increasing among younger individuals, becoming the primary cause of cancer deaths in men under 50.^[^ ^1^ ^]^ The incidence and mortality of CRC are particularly high in developed countries, largely due to factors such as lifestyle, diet, and genetics.^[^ ^2^ ^]^ Although advancements have been made in screening and treatment, early diagnosis of CRC remains challenging, often resulting in poor prognoses.^[^ ^3^ ^]^ Thus, further investigation into the pathogenesis of CRC, the identification of novel biomarkers, and the development of effective therapeutic strategies continue to be crucial areas of focus in cancer research.

Post‐translational modifications (PTMs) influence protein structure, function, interactions, stability, and localization, and are essential in driving the progression of CRC.^[^ ^4^ ^]^ Ubiquitination is a crucial post‐translational modification that primarily regulates protein stability. This process typically involves three ATP‐dependent steps mediated by E1, E2, and E3 ubiquitin ligases.^[^ ^5^, ^6^ ^]^ Among them, F‐box proteins (FBPs) have increasingly been recognized for their critical roles in tumor initiation and progression.^[^ ^7^, ^8^, ^9^ ^]^

F‐box only protein 44 (FBXO44), a member of the FBPs family, consists of two domains.^[^ ^10^ ^]^ The F‐box domain determines substrate specificity^[^ ^11^ ^]^ while the FBA domain mediates interaction with the substrate.^[^ ^12^, ^13^ ^]^ BRCA1 is the first reported substrate of FBXO44. FBXO44 binds to the N‐terminus of BRCA1, promoting its ubiquitination and degradation, thereby contributing to the progression of sporadic breast cancer.^[^ ^11^ ^]^ In addition, subsequent studies revealed that FBXO44 promotes the ubiquitination and degradation of regulator of G‐protein signaling 2 (RGS2),^[^ ^14^ ^]^ moreover, further studies revealed that the phosphorylation of Ser3 prevents RGS2 from binding to FBXO44, which in turn protects RGS2 from degradation by the proteasome.^[^ ^15^ ^]^ Recently, FBXO44 was shown to regulate pregnane X receptor (PXR) protein levels, influencing CYP3A4 expression and drug interactions.^[^ ^16^ ^]^ Overall, existing research has shown that FBXO44 is critically implicated in a variety of diseases. However, its role in CRC progression remains unclear.

Forkhead box protein P1 (FOXP1), belonging to the Forkhead‐box (FOX) family,^[^ ^17^ ^]^ is widely regarded as a tumor suppressor due to the positioning of the FOXP1 gene at the tumor suppressor locus 3p14.1.^[^ ^18^ ^]^ However, comprehensive studies have revealed that FOXP1 exhibits dual biological functions, acting as both a tumor suppressor and an oncogene.^[^ ^19^ ^]^ For example, FOXP1 has been reported to be highly expressed in diffuse large B‐cell lymphoma (DLBCL) and primary cutaneous large B‐cell lymphomas (PCLBCL), and is associated with poor prognosis.^[^ ^20^, ^21^, ^22^ ^]^ On the other hand, FOXP1 can inhibit the growth of pancreatic cancer by regulating the transcriptional activity of IRF1.^[^ ^17^ ^]^ Moreover, it has been reported that reducing FOXP1 expression enhances the transcription of cyclin E2, thereby accelerating the proliferation of breast cancer.^[^ ^23^ ^]^ FOXP1 has been described as acting as a tumor suppressor in colorectal cancer;^[^ ^24^ ^]^ however, its post‐translational regulation in colorectal cancer remains unclear. The mechanisms controlling FOXP1 protein stability, such as ubiquitin‐mediated degradation and coordination with other modifications, have not been defined. In addition, the E3 ubiquitin ligase responsible for FOXP1 degradation in CRC has not been identified, indicating a need for further investigation into its upstream regulatory mechanisms.

Our study revealed that FBXO44, an F‐box protein involved in ubiquitination, is significantly upregulated in CRC and is associated with poor prognosis. Mechanistically, we identified that FOXP1 interacts with FBXO44, and FBXO44 catalyzes K48‐linked polyubiquitination of FOXP1 at K377, promoting its proteasomal degradation. Moreover, we discovered that AURKA phosphorylates FOXP1 at Ser440, significantly reducing FOXP1 stability by enhancing its interaction with FBXO44. Our findings elucidate the interactions between ubiquitination and phosphorylation in the regulation of FOXP1 stability, positioning the FBXO44/FOXP1 pathway as a viable target for therapeutic strategies in CRC.

## 2 Results

### 2.1 FBXO44 is Highly Expressed in CRC Tissues and is Associated with Poor Prognosis

**Figure 1:**
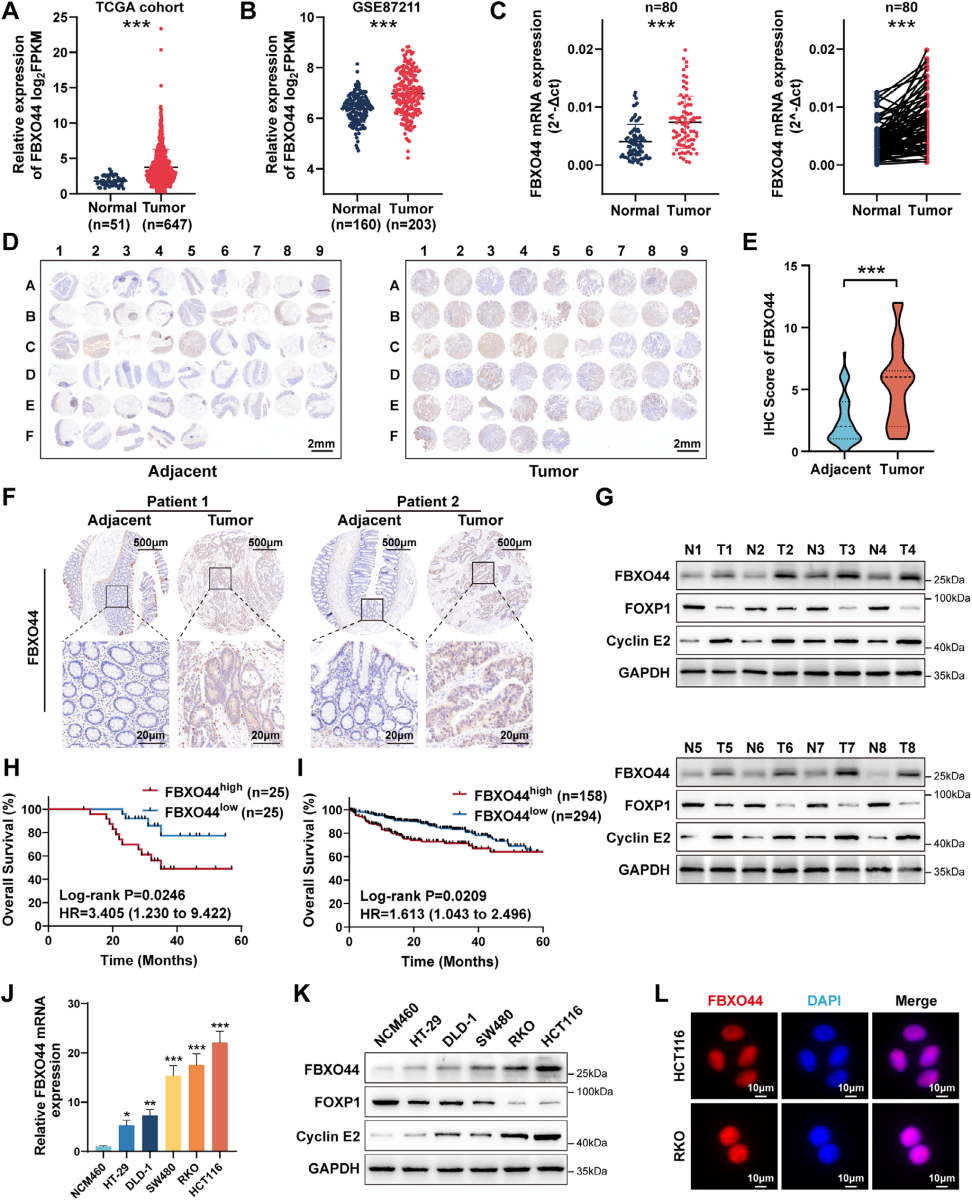
FBXO44 is highly expressed in CRC tissues and cell lines and is associated with poor prognosis. A) mRNA expression levels of FBXO44 based on TCGA datasets. B) mRNA expression levels of FBXO44 based on GSE87211. C) Quantification of FBXO44 mRNA levels in 80 CRC tissues and matched adjacent normal tissues by qRT‐PCR. D) Immunohistochemical (IHC) analysis of FBXO44 in a tissue microarray containing 50 cases of primary tumors and matched adjacent normal tissues. E) Violin plot showing IHC scores of FBXO44 in CRC TMA samples. F) Representative IHC images showing FBXO44 expression in matched CRC tumor and adjacent normal tissues. G) Western blot analysis of FBXO44, FOXP1, and Cyclin E2 protein levels in 8 paired CRC tumor and adjacent normal tissues. H) Kaplan–Meier survival analysis comparing patients with low (n=25) and high (n=25) FBXO44 expression based on CRC TMA. I) Kaplan‐Meier analysis of overall survival (OS) based on FBXO44 expression in patients from TCGA datasets. J) Relative mRNA expression levels of FBXO44 in NCM460 and CRC cell lines, as measured by qRT‐PCR. K) Western blot analysis of FBXO44, FOXP1, and Cyclin E2 protein levels in the indicated CRC cell lines and NCM460. L) Subcellular localization of FBXO44 in CRC cells. All data are presented as the means ± SD of three independent experiments. ^*^ *P* < 0.05, ^**^ *P* < 0.01, ^***^ *P* < 0.001.

To elucidate the role of FBXO44 in colorectal cancer (CRC) progression, we first examined its expression profile. We observed that FBXO44 expression was significantly upregulated in tumor tissues compared to normal tissues based on TCGA (https://tcga‐data.nci.nih.gov/) and GEO databases (GSE87211) (**Figure** **1**A,B). In our cohort of 80 paired CRC samples (cohort 1), FBXO44 mRNA levels were significantly elevated in tumor tissues (Figure 1C). Furthermore, analysis of a tissue microarray (TMA) comprising 50 paired CRC samples (cohort 2) from our center demonstrated a marked elevation of FBXO44 protein levels in tumor tissues, supported by higher IHC scores in these samples (Figure 1D–F). Additionally, samples from the TMA patient cohort were selected for WB analysis, confirming the elevated FBXO44 protein levels in CRC samples (Figure 1G). We analyzed the correlation between FBXO44 mRNA expression and clinical pathological features (cohort 1), finding significant positive associations with tumor size, T stage, and vascular invasion (Table S1, Supporting Information). Kaplan‐Meier survival analysis of the TMA cohort showed that high FBXO44 expression was linked to poorer overall survival in patients (Figure 1H). Correspondingly, survival analysis from the TCGA database indicated that patients with elevated FBXO44 expression also exhibited poorer overall survival (Figure 1I). We further assessed FBXO44 expression across a panel of CRC cell lines and observed that both protein and mRNA levels in NCM460 cells were significantly lower than in the tumor cell lines (Figure 1J,K). Immunofluorescence (IF) analysis of HCT116 and RKO cell lines demonstrated that FBXO44 is predominantly localized in the nucleus (Figure 1L). Collectively, these findings indicate that high expression of FBXO44 in CRC is linked to unfavorable prognosis.

### 2.2 FBXO44 Promotes CRC Cell Proliferation In Vitro

**Figure 2:**
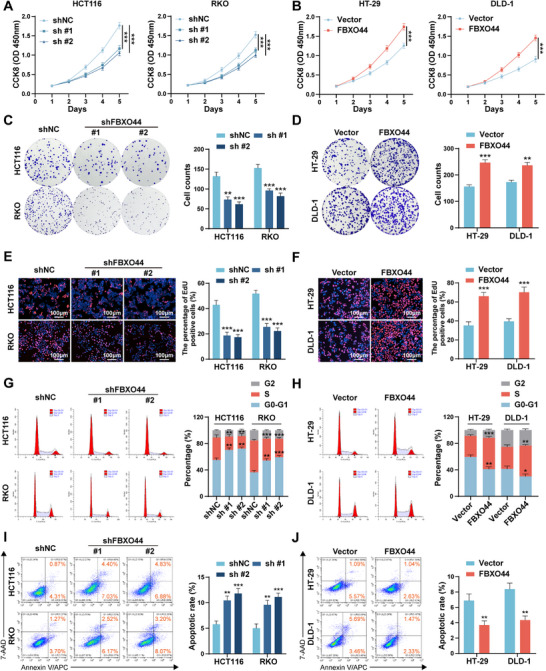
FBXO44 promotes CRC cell proliferation in vitro. A, B) CCK‐8 assays were utilized to determine the growth curves of cells with FBXO44 knockdown or overexpression. C, D) Colony formation assays were performed to assess the proliferation of CRC cells. E, F) EdU assays were conducted to evaluate cell proliferation ability. G, H) The cell cycle distribution was analyzed by flow cytometry in cells with FBXO44 knockdown or overexpression. I, J) Cells were cultured in serum‐free medium for 36 h, and apoptosis rates (LR+UR) were measured using flow cytometry. All data are presented as the means ± SD of three independent experiments. ^*^ *P* < 0.05, ^**^ *P* < 0.01, ^***^ *P* < 0.001.

To explore the role of FBXO44 in CRC, we knocked down FBXO44 in HCT116 and RKO cells, which exhibit higher FBXO44 expression, and overexpressed FBXO44 in HT‐29 and DLD‐1 cells, which have lower FBXO44 expression (Figure S1A, Supporting Information). Subsequently, CCK‐8, colony formation, and EdU assays were performed to assess the role of FBXO44 in the proliferation of CRC cells. The results indicated that knocking down FBXO44 markedly reduced the proliferation of HCT116 and RKO cells. Conversely, overexpressing FBXO44 in HT‐29 and DLD‐1 cells resulted in increased proliferation (**Figure** **2**A–F). Moreover, flow cytometry analysis revealed that knocking down FBXO44 inhibited the transition from G0‐G1 to S phase. However, overexpression of FBXO44 resulted in an increased proportion of cells in the S phase (Figure 2G,H). Apoptosis assays showed that knocking down FBXO44 increased the apoptotic rates of HCT116 and RKO cells. In contrast, overexpression of FBXO44 in HT‐29 and DLD‐1 cells markedly reduced the apoptotic rates (Figure 2I,J). Collectively, these findings indicate that FBXO44 enhances the proliferation of CRC cells in vitro.

### 2.3 FBXO44 Promotes CRC Cell Growth, Tumorigenesis In Vivo, and PDOs Formation

**Figure 3:**
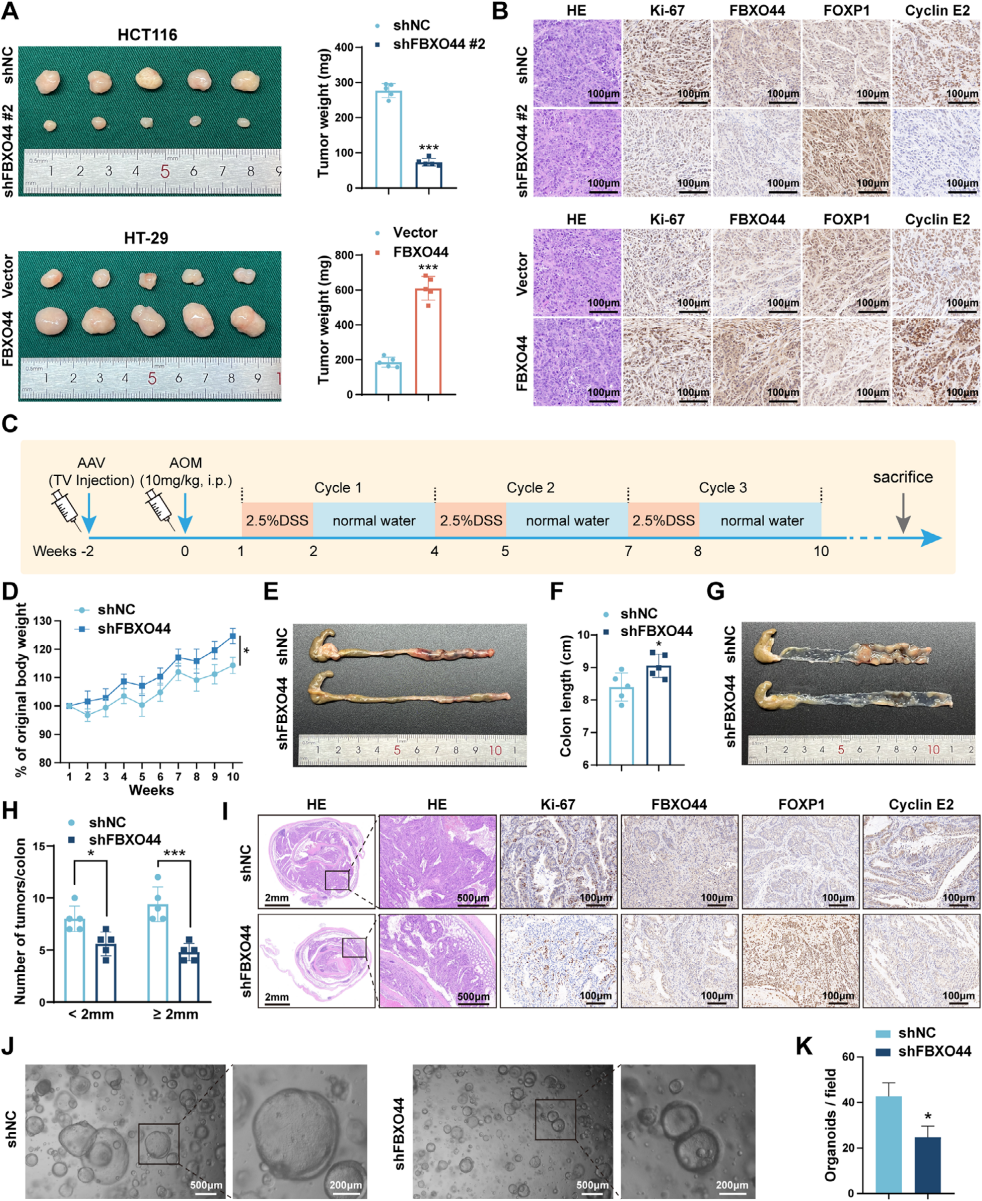
FBXO44 promotes CRC cell growth, tumorigenesis in vivo, and PDOs formation. A) Representative photographs of subcutaneous xenograft tumors from nude mice. Average tumor weight was measured at the endpoint. B) IHC was performed to determine the protein levels of Ki‐67, FBXO44, FOXP1, and Cyclin E2 in the xenograft tumors. C) Schematic diagram of AOM/DSS ‐induced CRC in BALB/c mice. D) The changes in the relative body weight of BALB/c mice over time are illustrated. E, F) Representative images of colons and colon lengths in different treatment groups of mice. G, H) Representative images of colons and the number of colonic tumors at least 2 mm or smaller than 2 mm per mouse. I) HE staining and IHC analysis of Ki‐67, FBXO44, FOXP1, and Cyclin E2 in tumors from different groups. J, K) FBXO44 was knocked down in human colorectal cancer organoids, and organoid number was observed. All data are presented as the means ± SD of three independent experiments. ^*^ *P* < 0.05, ^**^ *P* < 0.01, ^***^ *P* < 0.001.

To evaluate the in vivo role of FBXO44, we commenced by generating xenograft tumor models. Subcutaneous injections were administered to the respective groups of nude mice using HCT116 cells stably transfected with shNC or shFBXO44 #2, and HT‐29 cells stably transfected with Vector or FBXO44. The results indicated that knocking down FBXO44 inhibited tumor growth, leading to a decrease in tumor weight compared to the shNC group. Conversely, overexpression of FBXO44 produced the opposite effect (**Figure** **3**A). HE and IHC analyses demonstrated that Ki‐67 expression was significantly lower in the FBXO44 knockdown group relative to the shNC group, whereas the FBXO44 overexpression group showed a substantial increase in Ki‐67 levels (Figure 3B). Next, we examined a colon cancer (CAC) model induced by AOM/DSS in BALB/c mice (Figure 3C). Two weeks before inducing CAC, we injected adeno‐associated virus serotype 9 (AAV9) to modulate FBXO44 expression. The interference efficiency of FBXO44 was verified through WB (Figure S1B, Supporting Information). The results indicated that FBXO44 knockdown retarded the progression of CAC, as evidenced by enhanced body weight increase, decreased colon length, fewer tumor lesions, and less severe damage to colorectal tissues (Figure 3D–H). In contrast, overexpressing FBXO44 promoted CAC progression (Figure S1C–G, Supporting Information). HE and IHC analyses of mouse colorectal tissue revealed that Ki‐67 levels were lower in the shFBXO44 group than in the shNC group (Figure 3I), whereas the FBXO44 overexpression group exhibited higher Ki‐67 expression compared to the Vector group (Figure S1H, Supporting Information). We also generated patient‐derived organoids (PDOs) from CRC patient samples. FBXO44 knockdown in the PDOs resulted in a decrease in organoid number. (Figure 3J,K), overexpression of FBXO44, however, yielded the opposite results (Figure S1I, J, Supporting Information). Collectively, these experimental results confirm that upregulation of FBXO44 expression promotes tumorigenesis in vivo and organoid formation.

### 2.4 FBXO44 Interacts with FOXP1 and Promotes FOXP1 Degradation

**Figure 4:**
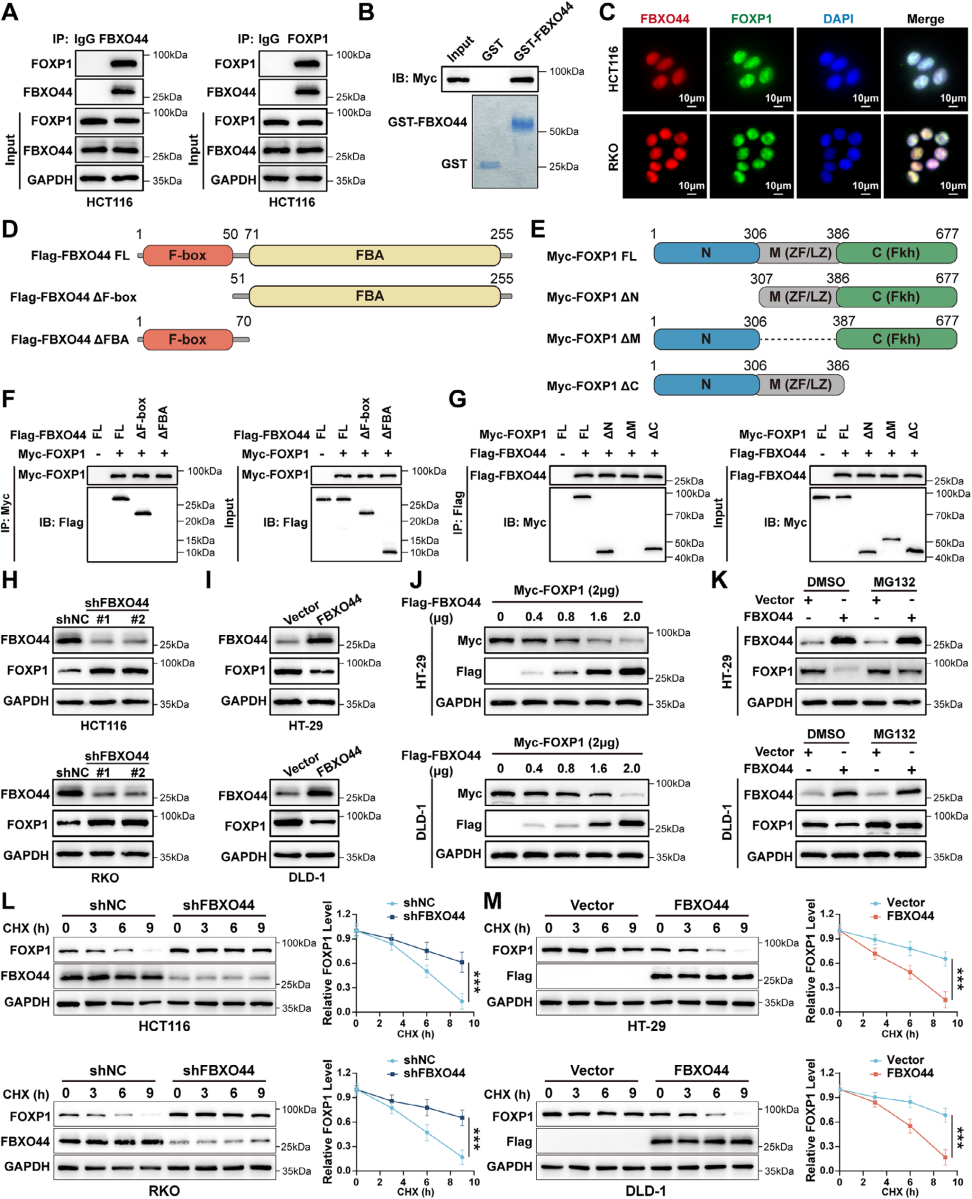
FBXO44 interacts with FOXP1 and promotes its degradation. A) HCT116 cells were treated with MG132 (20 µm) for 6 h, and cell lysates were subjected to immunoprecipitation followed by Western blot analysis using the indicated antibodies. B) Purified Myc‐FOXP1 was incubated with GST and GST‐FBXO44 coupled to glutathione‐Sepharose beads. The proteins retained on the Sepharose beads were analyzed by IB using the indicated antibodies. Recombinant GST‐FBXO44 was purified from bacteria and analyzed using SDS‐PAGE followed by Coomassie blue staining. C) Confocal microscopy showing the co‐localization of FBXO44 and FOXP1 in HCT116 and RKO cells. Nuclei were counterstained with DAPI. D, E) Schematic representation of full‐length (FL) Flag‐tagged FBXO44, Myc‐tagged FOXP1, and their respective deletion mutants. F) HEK293T cells were co‐transfected with Myc‐FOXP1 and FL Flag‐FBXO44 or deletion mutants. After treatment with MG132 (20 µm) for 6 h, cell lysates were subjected to immunoprecipitation followed by Western blot analysis using the indicated antibodies. G) HEK293T cells were co‐transfected with Flag‐FBXO44 and FL Myc‐FOXP1 or deletion mutants. After treatment with MG132 (20 µm) for 6 h, cell lysates were subjected to immunoprecipitation followed by Western blot assay with the indicated antibodies. H, I) FOXP1 protein levels were determined in HCT116 and RKO cells with FBXO44 knockdown, as well as in HT‐29 and DLD‐1 cells with FBXO44 overexpression. J) HT‐29 and DLD‐1 cells were transfected with plasmids encoding Myc‐FOXP1 and different concentrations of Flag‐FBXO44 for 24 h. Cell lysates were then analyzed by Western blot using the indicated antibodies. K) HT‐29 and DLD‐1 cells were transfected with either FBXO44 or an empty vector control and subjected to the indicated treatments. The expression levels of FOXP1 and FBXO44 were subsequently analyzed by Western blot. L) HCT116 and RKO cells transfected with either shNC or FBXO44 shRNAs were treated with CHX for the indicated durations. FOXP1 and FBXO44 protein levels were determined by Western blot analysis. Quantification of FOXP1 levels normalized to GAPDH is presented. M) Myc‐FOXP1 was co‐transfected with either vector control or Flag‐FBXO44 into HT‐29 and DLD‐1 cells. Following CHX treatment for the indicated durations, cell lysates were subjected to Western blot analysis. Quantification of FOXP1 levels normalized to GAPDH is presented. All data are presented as the means ± SD of three independent experiments. ^**^ *P* < 0.01, ^***^ *P* < 0.001.

To investigate the molecular mechanisms by which FBXO44 promotes CRC progression, we performed co‐immunoprecipitation (co‐IP) and mass spectrometry to identify proteins interacting with FBXO44. Among the top ten differential proteins identified, FOXP1 was the most abundant, suggesting that FOXP1 may be an interacting partner of FBXO44 (Figure S2A, B, Supporting Information). IHC analysis of tissue microarray from cohort 2 revealed that FOXP1 expression was reduced in CRC tissues compared to adjacent tissues (Figure S2C–E, Supporting Information). Kaplan‐Meier survival analysis showed that higher FOXP1 expression was associated with improved overall survival in CRC patients (Figure S2F, Supporting Information). Next, HDOCK SERVER analysis predicted that FBXO44 could interact with FOXP1 (Figure S2G, Supporting Information). Co‐immunoprecipitation assays were conducted to investigate the interaction between FBXO44 and FOXP1. The results indicated that endogenous FBXO44 interacts with FOXP1 (**Figure** **4**A), and this interaction was also observed with exogenously expressed proteins (Figure S2H, Supporting Information). Additionally, the GST pull‐down assay indicated that FBXO44 directly interacts with FOXP1 in vitro (Figure 4B). To investigate the subcellular distribution of FBXO44 and FOXP1, immunofluorescence staining was conducted in CRC cells. The findings indicated that FBXO44 and FOXP1 were primarily colocalized in the nucleus (Figure 4C). Proximity ligation assay (PLA) further confirmed that FBXO44 interacts with FOXP1 predominantly in the nucleus (Figure S2I, Supporting Information). Deletion mutants of FBXO44 and FOXP1 were generated to identify the critical domains for their interaction (Figure 4D,E). Co‐immunoprecipitation assays confirmed that the FBA domain of FBXO44 and the middle fragment of FOXP1 are necessary for their interaction (Figure 4F,G). We then investigated the influence of FBXO44 on FOXP1 at both the protein and mRNA levels. We found that knockdown of FBXO44 elevated FOXP1 protein levels in HCT116 and RKO cells (Figure 4H). Consistently, overexpression of FBXO44 reduced FOXP1 protein levels in HT‐29 and DLD‐1 cells (Figure 4I), and this regulation was dose‐dependent (Figure 4J). Notably, changes in FBXO44 expression did not affect FOXP1 mRNA levels (Figure S2J, Supporting Information), indicating post‐transcriptional regulation. We therefore hypothesized that FBXO44 facilitates FOXP1 ubiquitination and proteasomal degradation. To test this, CRC cells were treated with the proteasome inhibitor MG132, which significantly alleviated FOXP1 downregulation induced by FBXO44 overexpression (Figure 4K). Subsequently, we utilized cycloheximide (CHX) to block protein translation and found that FBXO44 knockdown significantly extended the half‐life of FOXP1 protein in HCT116 and RKO cells (Figure 4L). In contrast, overexpression of FBXO44 significantly reduced the half‐life of FOXP1 protein in HT‐29 and DLD‐1 cells (Figure 4M). Together, these data indicate FBXO44 interacts with FOXP1 and modulates FOXP1 protein levels at the post‐transcriptional level in CRC cells.

### 2.5 FBXO44 Catalyzes K48‐Linked Ubiquitination of FOXP1 at K377

**Figure 5:**
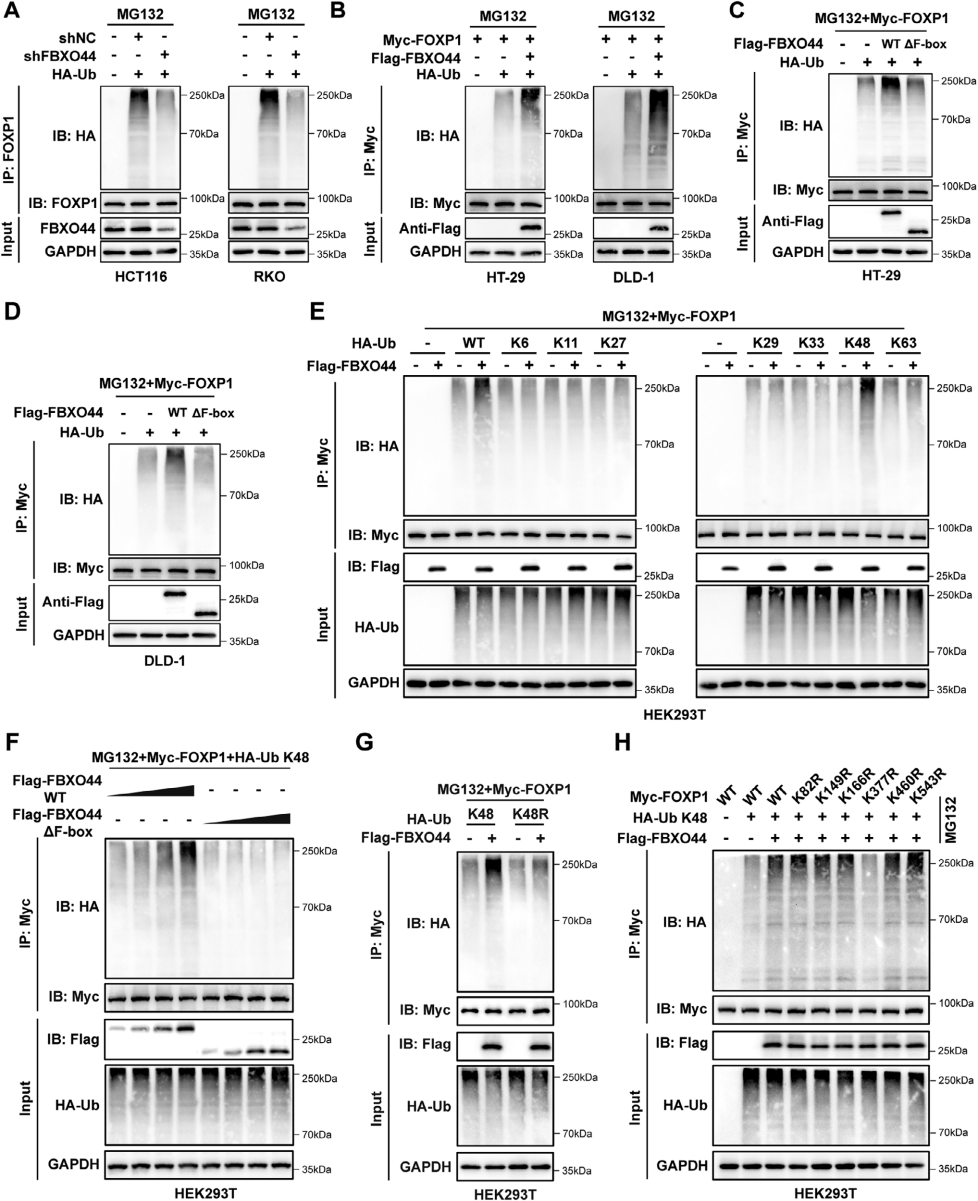
FBXO44 catalyzes K48‐linked ubiquitination of FOXP1 at K377. A) Ubiquitination levels of FOXP1 were determined by co‐immunoprecipitation in HCT116 and RKO cells transfected with FBXO44 shRNA. Cells were treated with MG132 (20 µm) for 6 h before collection. B) Ubiquitination levels of Myc‐FOXP1 were determined by co‐immunoprecipitation in HT‐29 and DLD‐1 cells transfected with Flag‐FBXO44. Cells were treated with MG132 (20 µm) for 6 h before collection. C, D) Ubiquitination levels of Myc‐FOXP1 were determined by co‐immunoprecipitation in HT‐29 and DLD‐1 cells transfected with either Flag‐FBXO44 or Flag‐FBXO44 ΔF‐box. Cells were treated with MG132 (20 µm) for 6 h before collection. E) HEK293T cells were co‐transfected with Flag‐FBXO44, Myc‐FOXP1, and either wild‐type ubiquitin or various ubiquitin mutants. After treatment with MG132 (20 µm) for 6 h, the cells were collected and subjected to co‐immunoprecipitation and western blot analysis. F) HEK293T cells were transfected with Myc‐FOXP1 and increasing amounts of either Flag‐FBXO44 WT or Flag‐FBXO44 ΔF‐box, and FOXP1 ubiquitination levels were subsequently analyzed. Cells were treated with MG132 (20 µm) for 6 h before collection. G) HEK293T cells were co‐transfected with Myc‐FOXP1, Flag‐FBXO44, and either wild‐type ubiquitin or the ubiquitin K48R mutant. After treatment with MG132 (20 µm) for 6 h, the cells were collected and subjected to co‐immunoprecipitation and western blot analysis. H) HEK293T cells were co‐transfected with Flag‐FBXO44, ubiquitin K48, and various Myc‐FOXP1 mutants, and FOXP1 ubiquitination levels were subsequently examined. Cells were treated with MG132 (20 µm) for 6 h before collection. All data are presented as the means ± SD of three independent experiments. ^**^ *P* < 0.01.

Next, we investigated whether FBXO44 downregulates FOXP1 via the ubiquitination pathway, as suggested by our previous findings. Given that FBXO44 is a component of the SCF complex, we first assessed the expression of its core catalytic subunit, CUL1, in CRC cell lines. Compared to NCM460 cells, CUL1 was consistently upregulated across CRC cell lines (Figure S3B, Supporting Information), and its protein levels were not altered by FBXO44 knockdown or overexpression (Figure S3C, Supporting Information). These findings suggest that FBXO44 may function in coordination with CUL1 as part of the SCF complex in CRC. Consistent with our hypothesis, we observed that FBXO44 knockdown decreased FOXP1 ubiquitination in HCT116 and RKO cells (**Figure** **5**A). Conversely, FBXO44 overexpression significantly increased FOXP1 ubiquitination in HT‐29 and DLD‐1 cells (Figure 5B). Previous reports indicate that the ubiquitination function of FBXO44 on its substrates is mediated by its F‐box domain.^[^ ^16^ ^]^ Consistent with this, our experiments showed that full‐length overexpression of FBXO44 increased FOXP1 ubiquitination, while FBXO44 lacking the F‐box domain (FBXO44 ΔF‐box) did not (Figure 5C,D). These results were independently validated by in vitro ubiquitination assays (Figure S3D, Supporting Information). To determine the specific ubiquitin chain type utilized by FBXO44 to enhance FOXP1 ubiquitination, we conducted co‐transfection experiments with linkage‐specific ubiquitin. Our findings revealed that FBXO44 mediates K48‐linked polyubiquitination of FOXP1 in a dose‐dependent manner (Figure 5E,F). Additionally, the ubiquitin mutant K48R, where lysine 48 is replaced with arginine (R), failed to increase the ubiquitination level of FOXP1 (Figure 5G). Furthermore, to identify the precise residue on FOXP1 that is modified by FBXO44, we used PhosphoSitePlus (https://www.phosphosite.org/homeAction.action) to predict several potential ubiquitination sites: K82, K149, K166, K377, K460, and K543. Based on these predictions, we generated a set of FOXP1 mutants and co‐expressed them with Flag‐FBXO44. Our results showed that FBXO44 failed to promote K48‐linked polyubiquitination of the FOXP1 K377R mutant (Figure 5H), as further confirmed by in vitro ubiquitination assays (Figure S3E, Supporting Information). Furthermore, the K377R mutant exhibits a prolonged protein half‐life in comparison to the wild‐type FOXP1 (Figure S3F, Supporting Information). Collectively, these data indicate that FBXO44 specifically catalyzes K48‐linked polyubiquitination of FOXP1 at K377, promoting its downregulation.

### 2.6 FBXO44 is Involved in the Development of Colorectal Cancer in a Manner Dependent on FOXP1

We further explored the functional significance of FBXO44‐mediated FOXP1 regulation on the phenotype of colorectal cancer. FBXO44‐depleted and control cells were transfected with lentiviral shRNA targeting FOXP1 or a control. Additionally, FBXO44, FOXP1, or corresponding vectors were transfected into CRC cells via lentivirus. Through the CCK‐8 assay, we observed that FOXP1 silencing mitigated the cell growth inhibition induced by FBXO44 deficiency. Conversely, the introduction of FOXP1 mitigated the growth‐promoting effects of FBXO44 overexpression (Figure S4A, B, Supporting Information). Comparable results were observed in colony formation and EdU staining assays (Figure S4C–F, Supporting Information). Flow cytometry analysis showed that FOXP1 deficiency reversed the G0‐G1 phase arrest and increased apoptosis rates caused by FBXO44 knockdown, and vice versa. (Figure S5A–D, Supporting Information). Additionally, xenograft tumor models indicated that FOXP1 deficiency reversed the suppression of subcutaneous tumor growth in nude mice induced by FBXO44 knockdown, and vice versa (Figure S6A–D, Supporting Information). Collectively, these findings suggest that FBXO44 promotes CRC proliferation primarily via FOXP1, both in vitro and in vivo.

### 2.7 AURKA‐Mediated Phosphorylation of FOXP1 Promotes its Ubiquitination and Degradation by FBXO44

**Figure 6:**
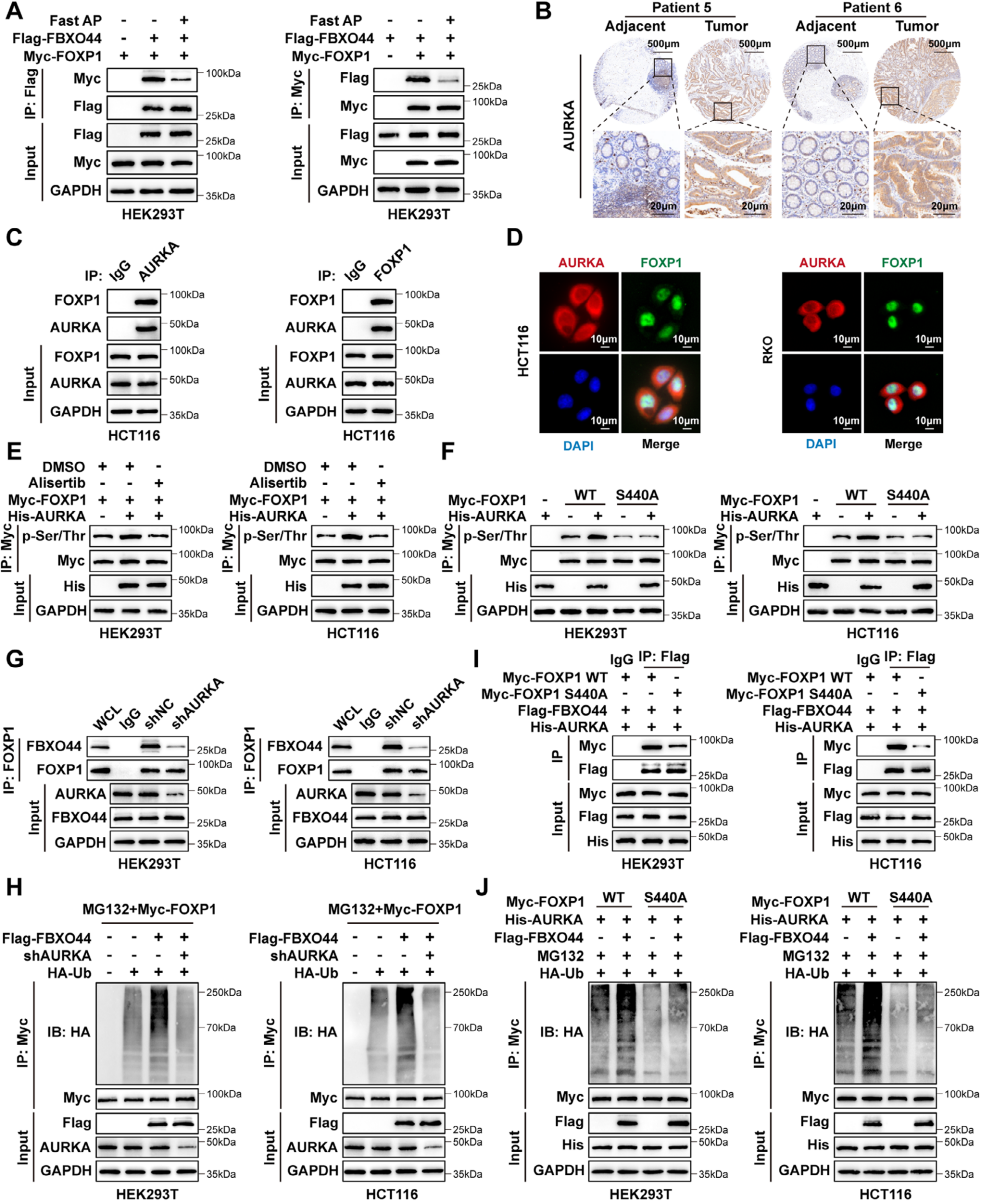
AURKA‐mediated phosphorylation of FOXP1 promotes its ubiquitination and degradation by FBXO44. A) HEK293T cells were transfected with the indicated plasmids for 24 h and subsequently treated with MG132 (20 µm) for 6 h. Cells were lysed with RIPA buffer (without SDS) and, where indicated, treated with Fast AP. The lysates were then subjected to immunoprecipitation using the indicated antibodies for analysis. B) Representative IHC images showing AURKA expression in matched CRC tumor and adjacent normal tissues. C) HCT116 cells were treated with MG132 (20 µm) for 6 h, and cell lysates were subjected to immunoprecipitation followed by Western blot analysis using the indicated antibodies. D) Confocal microscopy showing the co‐localization of AURKA and FOXP1 in HCT116 cells. Nuclei were counterstained with DAPI. E) Myc‐FOXP1 and His‐AURKA were co‐expressed in HEK293T and HCT116 cells, with or without Alisertib treatment. Cell lysates were subjected to IP using anti‐Myc antibody, followed by IB with antibodies against Myc, p‐Ser/Thr, and His. F) His‐AURKA and Myc‐FOXP1 WT or HA‐FOXP1 S440A mutant were co‐expressed in HEK293T and HCT116 cells. The cell lysates were subjected to IP using an anti‐Myc antibody, followed by IB with antibodies against Myc, p‐Ser/Thr, and His. G) HEK293T and HCT116 cells were transfected with either shNC or AURKA shRNAs. The cell lysates were then immunoprecipitated with anti‐FOXP1 antibody and subsequently analyzed. H) HEK293T and HCT116 cells were transfected with HA‐Ub, Flag‐FBXO44, or AURKA shRNAs and treated with MG132. Cell lysates were immunoprecipitated with anti‐FOXP1 antibody and immunoblotted with the indicated antibodies for the in vivo ubiquitination assay. I) HEK293T and HCT116 cells were transfected with His‐AURKA, Flag‐FBXO44, and either Myc‐FOXP1 WT or the phosphorylation‐deficient Myc‐FOXP1 S440A mutant. Cell lysates were immunoprecipitated with anti‐Flag antibody and subsequently analyzed. J) HEK293T and HCT116 cells were transfected with His‐AURKA, HA‐Ub, Flag‐FBXO44, Myc‐FOXP1 WT or phosphorylation‐deficient Myc‐FOXP1 S440A mutant, and treated with MG132. Cell lysates were immunoprecipitated with anti‐Myc antibody and immunoblotted with the indicated antibodies for the in vivo ubiquitination assay.

Given that F‐box proteins are typically known to recognize phosphorylated substrates, we conducted a Co‐IP experiment following Fast Alkaline Phosphatase (Fast AP) treatment to assess if FBXO44 interacts with FOXP1 in a phosphorylation‐dependent way. Our results showed that the interaction between FBXO44 and FOXP1 was disrupted after Fast AP treatment (**Figure** **6**A), indicating that phosphorylation is crucial for this interaction. Previous reports have shown that FOXP1 is a phosphorylation substrate of Aurora kinase A (AURKA).^[^ ^25^ ^]^ Therefore, we aimed to investigate whether the ubiquitination of FOXP1 by FBXO44 depends on the phosphorylation of FOXP1 by AURKA. First, IHC analyses revealed that AURKA is markedly elevated in the majority of CRC patients (Figure 6B and Figure S7A,B, Supporting Information). Both endogenous and exogenous co‐immunoprecipitation experiments demonstrated mutual co‐precipitation between FOXP1 and AURKA (Figure 6C and Figure S7C, Supporting Information). Immunofluorescence analysis revealed predominant nuclear co‐localization of AURKA and FOXP1 (Figure 6D), which was further validated by PLA (Figure S7D, Supporting Information). We observed that overexpression of AURKA leads to increased phosphorylation of FOXP1, whereas treatment with the AURKA inhibitor Alisertib results in reduced phosphorylation of FOXP1 (Figure 6E). Next, we aimed to identify potential phosphorylation sites of FOXP1 by AURKA. By analyzing the amino acid sequence of FOXP1, we discovered that it contains an AURKA‐binding motif (R/K/N‐R‐X‐S/T‐B), which includes a phosphorylation site at S440 (Figure S7E, Supporting Information), consistent with previous mass spectrometry results.^[^ ^25^ ^]^ Experimental results show that the phosphorylation‐deficient mutant Ala (A) (S440A), which substitutes Ser440 with Alanine (A), abolishes the phosphorylation of FOXP1 (Figure 6F). Additionally, FOXP1 (S440A) with reduced phosphorylation has a significantly longer half‐life, while the phosphorylation‐mimetic FOXP1 (S440D) notably decreases FOXP1 stability (Figure S7F, Supporting Information). Knocking down AURKA markedly suppresses the interaction between FOXP1 and FBXO44, thus further substantiating our hypothesis (Figure 6G). Furthermore, the downregulation of AURKA significantly extended the half‐life of FOXP1 in CRC cells that overexpress FBXO44 (Figure S7G, Supporting Information). We further investigated the impact of AURKA‐mediated phosphorylation on FOXP1 ubiquitination. Notably, knocking down AURKA significantly suppressed FBXO44‐induced ubiquitination of FOXP1 (Figure 6H). Lastly, we explored whether phosphorylation of FOXP1 at Ser440 affects its interaction with AURKA‐mediated FOXP1 and FBXO44. Our results indicate that the S440A mutation, which abolishes FOXP1 phosphorylation, notably reduces the AURKA‐enhanced interaction between FOXP1 and FBXO44 (Figure 6I). Furthermore, we observed that AURKA fails to enhance the ubiquitination of the S440A phospho‐defective mutant of FOXP1 (Figure 6J). These findings indicate that phosphorylation of FOXP1 at Ser440 by AURKA enhances its interaction with FBXO44, thereby decreasing FOXP1 stability in colorectal cancer.

### 2.8 The AURKA Inhibitor Alisertib can Reverse Colorectal Cancer Progression Driven by FBXO44 Overexpression

**Figure 7:**
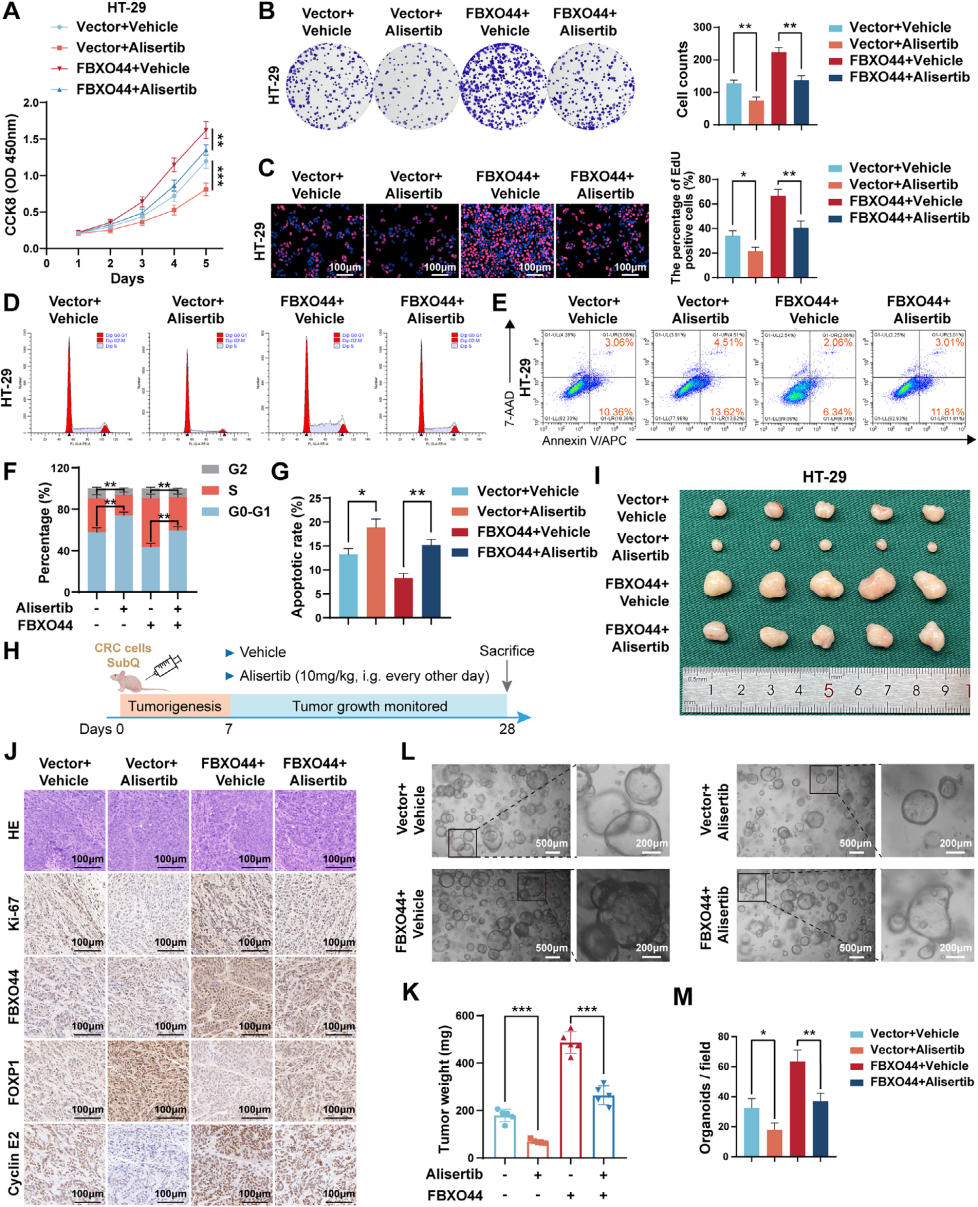
The AURKA inhibitor Alisertib can reverse colorectal cancer progression driven by FBXO44 overexpression. A–C) CCK‐8, colony formation, and EdU assays were performed on HT‐29 cells that were transfected with the indicated plasmids and subsequently treated with or without Alisertib. D–G) Flow cytometry analyses of the cell cycle and apoptosis were conducted on HT‐29 cells transfected with the indicated plasmids, followed by treatment with or without Alisertib. H) Schematic design for HT‐29 subcutaneous tumor model. I, K) Representative photographs show subcutaneous xenograft tumors in nude mice from different treatment groups. Average tumor weight was measured at the endpoint. J) HE and IHC staining of xenograft tumors. The expression of Ki‐67, FBXO44, FOXP1, and Cyclin E2 was detected by IHC. L, M) Representative images of human colorectal cancer organoids from different treatment groups, along with observations of organoid number. All data are presented as the means ± SD of three independent experiments. ^*^ *P* < 0.05, ^**^ *P* < 0.01, ^***^ *P* < 0.001.

Alisertib, also known as MLN8237, is a small molecule inhibitor that primarily targets AURKA. Next, we aim to further investigate whether Alisertib can reverse the promoting effect of FBXO44 in CRC. Alisertib effectively inhibited the enhanced cell growth induced by FBXO44 overexpression, as demonstrated by results from the CCK‐8 assay (**Figure** **7**A). Similar results were obtained in colony formation and EdU staining assays (Figure 7B,C). Moreover, Alisertib induces G0‐G1 phase arrest and enhances apoptosis rates following FBXO44 overexpression (Figure 7D–G). In a xenograft model involving nude mice, upregulation of FBXO44 notably increased tumor growth, leading to increased tumor weight and Ki‐67 expression. Importantly, this promoting effect is notably reversed by Alisertib (Figure 7H–K). No coincidence, we discovered that Alisertib can counteract the promoting effect of FBXO44 on colorectal cancer organoid growth (Figure 7L,M). These results demonstrate that Alisertib can reverse colorectal cancer progression driven by overexpression of FBXO44.

### 2.9 FBXO44 Enhances Cyclin E2 Transcriptional Activity by Promoting the Degradation of FOXP1

**Figure 8:**
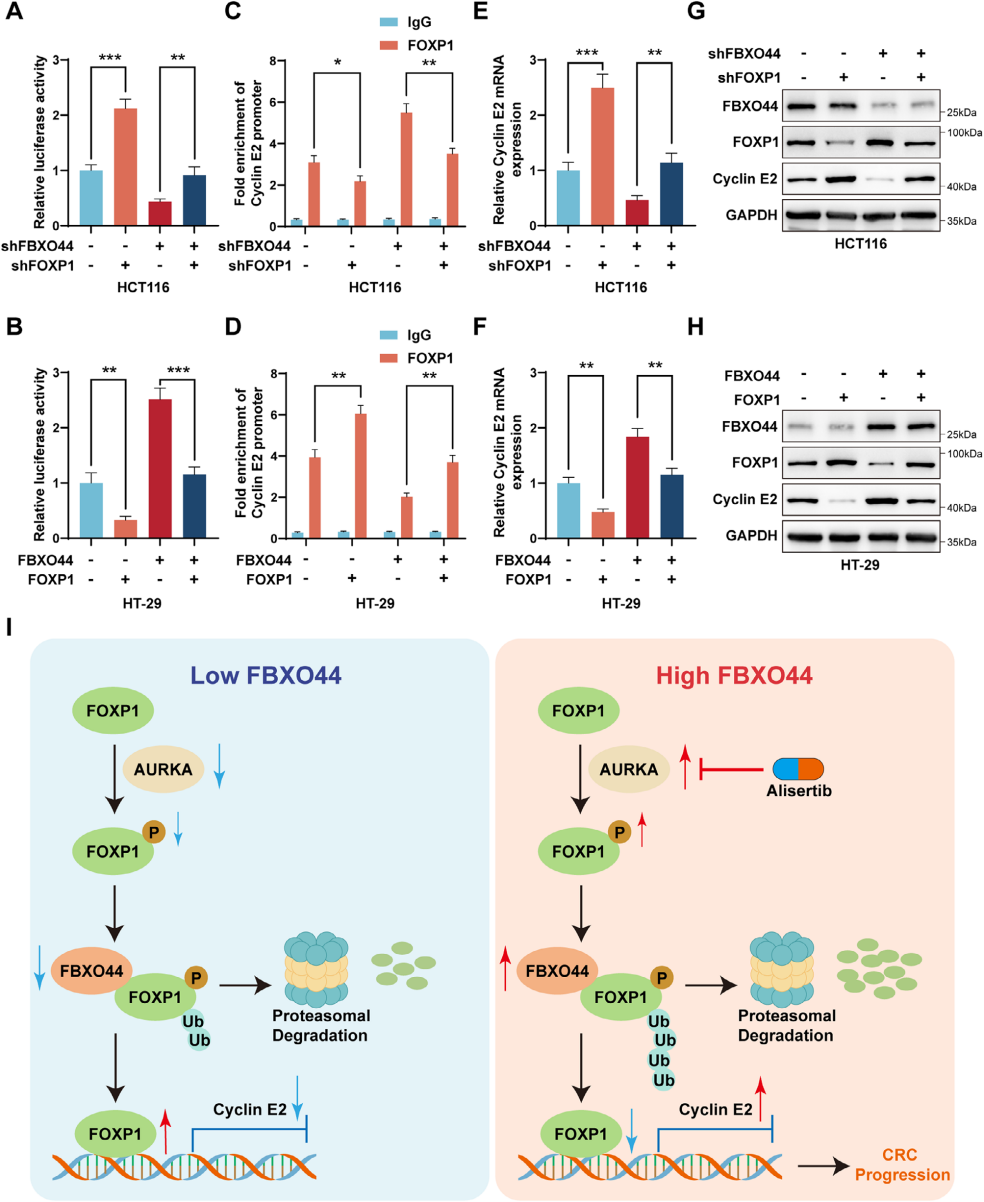
FBXO44 enhances Cyclin E2 transcriptional activity by promoting the degradation of FOXP1. A, B) Luciferase reporter assays were employed to assess the transcriptional activity of Cyclin E2 in the indicated cells. C, D) ChIP‐qPCR assays were utilized to assess the binding affinity of FOXP1 to the Cyclin E2 promoter in the indicated cells. E–H) The mRNA and protein expression levels of Cyclin E2 were measured using qRT‐PCR and Western blot analysis in the indicated cells. I) A schematic model for the mechanisms of FBXO44 in CRC. All data are presented as the means ± SD of three independent experiments. ^*^ *P* < 0.05, ^**^ *P* < 0.01, ^***^ *P* < 0.001.

Since it has been reported that FOXP1 inhibits the transcription of Cyclin E2,^[^ ^23^ ^]^ we aimed to investigate whether FOXP1 exerts a similar effect in CRC. Consistent with our hypothesis, luciferase reporter assays indicated that knockdown of FOXP1 resulted in an increase in the transcriptional activity of Cyclin E2, while overexpression of FOXP1 led to a decrease (**Figure** **8**A,B). ChIP‐qPCR analysis demonstrated that knockdown of FOXP1 reduced its binding to the Cyclin E2 promoter, whereas overexpression of FOXP1 enhanced this interaction (Figure 8C,D). Furthermore, qRT‐PCR and Western blot analyses revealed that FOXP1 knockdown elevated both mRNA and protein levels of Cyclin E2, in contrast to the effects observed with FOXP1 overexpression (Figure 8E–H).

Considering that FBXO44 promotes the ubiquitin‐mediated degradation of FOXP1, we further investigated whether FBXO44 regulates Cyclin E2 transcription through FOXP1 in CRC. We found that knockdown of FOXP1 effectively reversed the reduction in Cyclin E2 transcriptional activity induced by FBXO44 knockdown, and conversely, overexpression of FOXP1 reversed the effects of FBXO44 overexpression (Figure 8A,B). Furthermore, the knockdown of FOXP1 also mitigated the increase in FOXP1 binding to the Cyclin E2 promoter resulting from FBXO44 knockdown, whereas overexpression of FOXP1 yielded the opposite outcome (Figure 8C,D). Additionally, knockdown of FOXP1 reversed the downregulation of Cyclin E2 expression mediated by FBXO44 knockdown, while overexpression of FOXP1 demonstrated an opposing effect (Figure 8E–H).

Moreover, we found that, compared to NCM460, the protein levels of FBXO44 and Cyclin E2 were elevated, whereas FOXP1 protein levels were reduced in CRC cell lines. Similarly, tumor tissues exhibited higher FBXO44 and Cyclin E2 protein expression and lower FOXP1 protein expression compared to adjacent normal tissues. These findings indicate a positive correlation between FBXO44 and Cyclin E2 protein levels, and a negative correlation with FOXP1 (Figure 1G,K). Consistent protein expression patterns were also observed in both subcutaneous xenograft tumors and AOM/DSS‐induced CRC models (Figure 3B,I; Figure 7J; Figure S1H; Figure S6C,D, Supporting Information).

Collectively, these findings suggest that FBXO44 is essential for regulating Cyclin E2 transcription by modulating FOXP1 expression.

## 3 Discussion

FBXO44 has been reported to act as a substrate adaptor in SCF E3 ligase complexes during the progression of various diseases.^[^ ^14^, ^15^, ^16^ ^]^ Additionally, it has been shown to inhibit repetitive DNA elements in cancer cells through the recruitment of chromatin modifiers.^[^ ^26^ ^]^ However, the function of FBXO44 in CRC is not fully understood. In our study, FBXO44 levels were notably elevated in CRC tissues relative to adjacent normal tissues. CRC patients with increased FBXO44 exhibited reduced overall survival compared to those with lower expression. Elevation of FBXO44 significantly promoted CRC cell proliferation, whereas its downregulation notably inhibited it. Furthermore, our study elucidates the mechanism by which phosphorylation and ubiquitination regulate FOXP1 degradation to promote colorectal cancer progression. We identified FBXO44 as a binding protein that mediates the ubiquitin‐dependent degradation of FOXP1. Importantly, AURKA‐induced phosphorylation at Ser440 of FOXP1 enhances the interaction between FBXO44 and FOXP1, leading to increased ubiquitination and decreased stability of FOXP1. This process lifts FOXP1's transcriptional repression of Cyclin E2, thereby promoting CRC progression (Figure 8I). These findings highlight FBXO44 as a potential therapeutic target in CRC.

F‐box proteins are essential parts of the SCF ubiquitin ligase complex. They bind directly to substrates through specific domains, leading to ubiquitin‐mediated degradation of these proteins.^[^ ^27^ ^]^ F‐box proteins are crucial in the initiation and advancement of cancer.^[^ ^7^ ^]^ One key feature of the F‐box protein family is their ability to specifically recognize phosphorylated substrates, mediating their polyubiquitination and targeting them for proteasomal degradation.^[^ ^28^, ^29^ ^]^ Among these, FBXW7 is one of the most extensively studied F‐box proteins, known for targeting substrates like c‐Myc, YAP, p53, and PD‐1 in a phosphorylation‐dependent manner to promote their ubiquitination and degradation.^[^ ^30^, ^31^, ^32^, ^33^, ^34^ ^]^ Similarly, FBXL6 has been reported to target phospho‐p53 (S315), promoting its polyubiquitination and proteasomal degradation, which in turn suppresses p53 signaling in CRC.^[^ ^35^ ^]^ Additionally, the ubiquitination and degradation of Snail1 by FBXO31 depend on its phosphorylation by protein kinase D1 (PKD1), which is essential for FBXO31 to recognize and bind Snail1.^[^ ^36^ ^]^ Furthermore, FBXW2 binds to β‐catenin via EGF‐AKT1‐mediated phosphorylation at Ser552, promoting its ubiquitination and degradation, thereby inhibiting lung cancer by blocking β‐catenin‐driven transactivation of MMPs.^[^ ^37^ ^]^ Consistent with the characteristics of the aforementioned F‐box protein family, our study found that FBXO44 works synergistically with AURKA, which enhances the binding of FBXO44 to FOXP1 by promoting the phosphorylation of FOXP1 at Ser440, thereby accelerating FOXP1 degradation. This mechanism lifts FOXP1's transcriptional repression of Cyclin E2, thus promoting the development and progression of CRC.

On another note, kinases play an indispensable role in the crosstalk between phosphorylation and ubiquitination. For instance, AMPK has been reported to phosphorylate PROX1 at Ser79, which enhances the recruitment of the CUL4‐DDB1 ubiquitin ligase, thereby promoting PROX1 degradation.^[^ ^38^ ^]^ Similarly, GSK3β phosphorylates FASN at Thr827 and Ser831, leading to its destabilization through enhanced FBXW7β‐mediated ubiquitination and degradation.^[^ ^39^ ^]^ Additionally, DNA damage activates ATM, resulting in the phosphorylation of p53 at Ser33 and Ser37, which facilitates the binding of FBXW7 and the subsequent degradation of p53 by the SCF^FBXW7^ complex.^[^ ^33^ ^]^ In our study, we found that the kinase AURKA enhances the interaction between FOXP1 and FBXO44 by phosphorylating FOXP1 at the Ser440 site. This leads to increased ubiquitination and reduced stability of FOXP1 in CRC. AURKA is known to regulate cell cycle progression, mitosis, and essential oncogenic signaling pathways in numerous malignancies.^[^ ^25^ ^]^ Interestingly, earlier research has indicated that AURKA is also involved in the crosstalk between ubiquitination and phosphorylation. For instance, AURKA phosphorylates p53 at Ser315, which results in its ubiquitination by Mdm2 and subsequent proteolytic degradation.^[^ ^40^ ^]^ Similarly, our research found that AURKA has a comparable function in CRC.

Honestly, our research also has some limitations. First, while we confirmed that FBXO44 promotes CRC proliferation, its function in CRC metastasis and additional malignant characteristics is still not fully understood. Secondly, exploring the regulation of FBXO44 expression and activity in CRC cells could uncover novel regulatory mechanisms and identify upstream factors that control its oncogenic functions. Thirdly, further investigation is required to clarify the specific molecular mechanisms involved in FBXO44‐mediated ubiquitination, as well as to investigate the therapeutic potential of FBXO44 inhibitors in CRC. We will address these questions in future experiments.

In conclusion, this research offers important perspectives on the role of FBXO44 in CRC progression, emphasizing its potential as a therapeutic target and biomarker. By elucidating the mechanisms of FBXO44‐mediated FOXP1 degradation and its impact on CRC cell proliferation, we pave the way for future research to develop targeted therapies that can effectively inhibit CRC progression and improve patient outcomes.

## 4 Experimental Section

### Human Specimens and Cell Lines

Samples were procured from patients in the General Surgery Department of the First Affiliated Hospital of Nanjing Medical University, with informed consent obtained from all participants. Freshly excised tissues were rapidly frozen at −80 °C to ensure optimal preservation. All patients had not received neoadjuvant chemoradiotherapy prior to their surgical procedures.

The cell lines (HT‐29, DLD‐1, SW‐480, RKO, HCT116, NCM460, and HEK293T) were sourced from the Cell Bank of the Chinese Academy of Sciences (Shanghai, China). All cell lines were confirmed to be free of mycoplasma contamination and were maintained in a humidified incubator at 37 °C with 5% CO2.

### Western Blot Analysis

Western blot analysis was performed according to the procedures outlined in our previous study.^[^ ^41^ ^]^ Details of the antibodies used are provided in Table S2 (Supporting Information).

### Immunohistochemistry (IHC)

IHC was conducted following the methods described in previous studies.^[^ ^42^ ^]^ FBXO44, FOXP1 and AURKA expression levels were calculated by multiplying the proportion and intensity scores, as detailed in prior research.^[^ ^43^ ^]^ The primary antibodies used are listed in Table S2 (Supporting Information).

### Cell Proliferation Assays

Cell proliferation was evaluated through CCK‐8, colony formation, and EdU assays, employing methodologies outlined in previous research.^[^ ^44^ ^]^

### Flow Cytometry Assays

Flow cytometry was employed to assess cell cycle distribution and apoptosis rates in CRC cells, following the procedures previously described.^[^ ^45^ ^]^

### Co‐Immunoprecipitation (Co‐IP) Assay

Cell lysates were incubated overnight at 4 °C with a specific primary antibody, followed by the addition of A/G magnetic beads for 1 h. The beads were then washed twice with IP buffer and once with pure water. Next, samples were mixed with 1× SDS loading buffer and heated for 10 min. Immunoprecipitated proteins were analyzed by western blotting or mass spectrometry (BGI Shenzhen, Guangdong, China). To avoid interference from IgG heavy chains in immunoblotting, a light‐chain specific secondary antibody (CST #93 702) was used. Details of the primary antibodies are provided in Table S2 (Supporting Information).

### In Vivo Ubiquitination Assay

Cells subjected to specific treatments were treated with the proteasome inhibitor MG132 (Beyotime, Shanghai, China) for 6 h before collection. Subsequently, co‐immunoprecipitation was performed on the cell lysates using an anti‐Myc antibody, and the ubiquitination levels of FOXP1 were assessed using an anti‐HA antibody.

### Organoids Construction

Fresh tumor tissues were washed with cold PBS containing penicillin‐streptomycin, minced into 3–5 mm pieces, and digested with 5 mm EDTA on ice for 60 min. After observing cell clumping under a microscope, the cells were mixed with Matrigel and seeded into 24‐well plates. After 24 h of incubation, the organoids were subjected to lentiviral infection for 6 h, followed by medium replacement every three days.

### Luciferase Reporter Assay

CRC cells were co‐transfected with a luciferase reporter plasmid containing the Cyclin E2 promoter region and the corresponding plasmids or shRNAs. Subsequently, luciferase activity was quantified using the Dual‐Luciferase reporter kit (Promega, USA), following the previously described protocol.^[^ ^46^ ^]^

### Chromatin Immunoprecipitation Assays (ChIP)

ChIP assays were conducted using the ChIP Kit (#56 383, CST) in accordance with the manufacturer's instructions to evaluate protein‐DNA interactions. After immunoprecipitation, the purified DNA was analyzed by qPCR. The specific primers utilized for detecting the target regions are provided in Table S3 (Supporting Information).

### Animal Models

Five‐week‐old male BALB/c nude mice were employed to establish xenograft models. Each mouse received subcutaneous injections of 1 × 10^6^ transfected CRC cells in the left and right inguinal regions. Tumor volumes were assessed weekly. After a 28‐day period, the mice were euthanized, and the xenograft tumors were excised, weighed, and prepared for H&E and IHC staining.

The construction method for the AOM/DSS model of colorectal tumorigenesis was detailed in prior studies^[^ ^47^ ^]^ two weeks prior to the initiation of modeling, an adeno‐associated virus serotype 9 (AAV9) was administered via tail vein injection to disrupt FBXO44 expression. Throughout the experiment, the mice were monitored for clinical symptoms and body weight changes. At the conclusion of the study, the mice were euthanized, and their colons were collected for histopathological analysis to assess tumor development and other pathological alterations. All animal experiments were reviewed and approved by the Animal Ethics Committee of Nanjing Medical University.

### Statistical Analysis

Each experiment was conducted at least three times. GraphPad Prism software (La Jolla, CA, USA) and SPSS 15.0 software (Chicago, IL, USA) were used for statistical analyses. Statistical comparisons in this study were conducted using the Student's *t*‐test, ANOVA, Chi‐square test, and Kaplan‐Meier analysis. The significance level for all tests was set at 0.05.

### Ethics Statement

Approval for the human tissue study was obtained from the ethics committee of The First School of Clinical Medicine, Nanjing Medical University, and informed consent was acquired from all participating patients. All animal experiments were conducted in accordance with the guidelines set by the Committee on the Ethics of Animal Experiments at Nanjing Medical University, under the ethical approval number IACUC‐2310059. All procedures adhered to the relevant ethical guidelines and regulations.

## Conflict of Interest

The authors declare no conflict of interest.

## Author Contributions

H.N., H.X., S.Y., C.T. and T.W. contributed equally to this work. C. T. is a co‐first author. H.N., Y.S., Y.F., and J.T. were responsible for the overall conception and design of the research. H.N., H.X., C.T. and T.W. performed molecular experiments. C.T., C.J. and Z.C. designed and implemented the animal experiments. S.Y. analyzed the experimental results and provided bioinformatics support. H. N. authored the manuscript. Y.S., Y.F., J.T., and X.W. provided overall supervision of the research, secured funding, and contributed to the interpretation of the results. All authors read and approved the final manuscript.

## Supporting information



Supporting Information

Supplemental Table 1

Supplemental Table 2

Supplemental Table 3

## Data Availability

The data that support the findings of this study are available from the corresponding author upon reasonable request.
