# Supplementary material for: FBXO44 Regulates FOXP1 Degradation Through AURKA‐Dependent Phosphorylation to Promote Colorectal Cancer Progression
Source: Adv Sci (Weinh). 2025 Oct 6;12(47):e15458. doi: 10.1002/advs.202415458 (PMC12713037; doi:10.1002/advs.202415458)
Supplement: Supplementary file 1 — Supporting Information [file ADVS-12-e15458-s004.docx]

**Quantitative real-time PCR (qRT-PCR)**

**Total RNA of CRC cells and tissues was extracted using FreeZol Reagent (Vazyme, Jiangsu, China). Purified RNA was reverse transcribed into complementary DNA (cDNA) using HiScript III RT SuperMix for qPCR (Vazyme, Jiangsu, China). Subsequently, quantitative real-time PCR (qRT-PCR) was performed utilizing the SYBR Premix Ex Taq Kit (TaKaRa Biotechnology, Dalian, China). The primers for these transcripts are listed in Table S3.**

**Immunofluorescence (IF)**

**Cells were fixed with 4% paraformaldehyde, washed thrice with PBS, and permeabilized using 0.1% Triton X-100 in PBS. After overnight incubation with primary antibodies at 4°C, cells were exposed to secondary antibodies for two hours at room temperature in darkness. Nuclei were visualized using DAPI staining. Fluorescence images were acquired using the Stellaris STED laser scanning confocal microscopy system (LEICA, Germany). Details on the antibodies utilized can be found in Table S2.**

**Molecular Docking**

**Structural models of the receptor protein FOXP1 (Uniprot ID: Q9H334) and the ligand protein FBXO44 (Uniprot ID: Q9H4M3) were obtained from the Uniprot database. Preprocessing steps, including removal of water molecules and redundant ligands as well as hydrogen atom addition, were performed using PyMOL (version 2.4). Protein–protein docking was carried out using the HDOCK SERVER (http://hdock.phys.hust.edu.cn/). Docking Score, Confidence Score, and Ligand RMSD were employed as evaluation metrics. The model with the highest docking score was selected as the optimal docking conformation. Protein–protein interactions were visualized using PyMOL.**

**Proximity Ligation Assay (PLA)**

**HCT116 cells were plated onto confocal dishes and cultured overnight to ensure proper attachment. Cells were then fixed with 4% paraformaldehyde at 37 °C for 60 minutes, followed by permeabilization and blocking. After incubation with primary antibodies at 4 °C overnight, PLA reactions were carried out using the NavinciFlex Cell MR Kit (Navinci, Atto647N), according to the manufacturer's instructions. This included probe binding, ligation, and rolling circle amplification. Nuclei were counterstained with DAPI, and PLA signals were visualized using a Thunder Imager rapid high-resolution inverted fluorescence microscope (Leica).**

**Cell transfection**

**Cell transfection in this study primarily involved lentiviral shRNA and plasmid transfections. All transfections were performed using Lipo3000 transfection reagent (Beyotime, Shanghai, China) according to the manufacturer's protocol. Transfection efficiency was validated using qRT-PCR and Western blotting. The lentiviral shRNAs (Obio, Shanghai, China) used in this study are listed in Table S3.**

**GST pull-down assay**

**Myc-FOXP1 protein was purified from HEK293T cells, while recombinant GST-FBXO44 was purified from E. coli. GST pull-down assays were conducted at 4 °C, with GST and GST-FBXO44 immobilized on glutathione agarose resin using Myc-FOXP1. The pull-down complexes were washed five times, then boiled in SDS loading buffer for 10 minutes. The samples were subjected to SDS-PAGE and analyzed by immunoblotting using specific antibodies.**

**In vitro ubiquitination assay**

**Purified SCF^FBXO44^ complex was incubated with E1 UBE1, E2 UbcH5c, His-tagged ubiquitin (WT or mutant), ATP, and GST-FOXP1 (WT or K377R). After the reaction, Ni-NTA pull-down was performed, and bound proteins were analyzed by immunoblotting.**

**
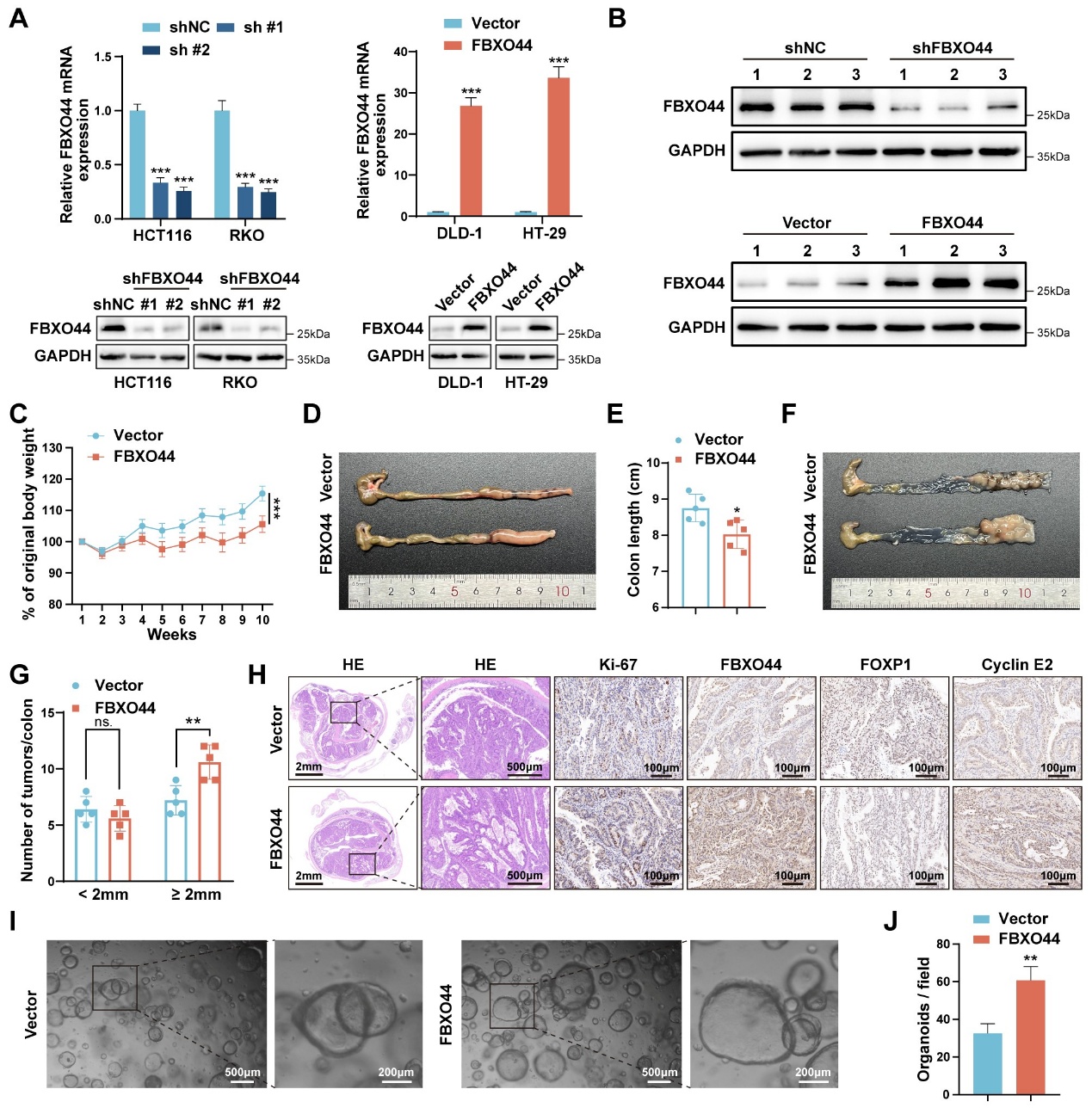
**

**Fig. S1 The construction of FBXO44 stably knockdown and overexpression CRC cell lines, and overexpressing FBXO44 promoted CAC progression.**

**A.** qRT-PCR and western blotting were performed to evaluate the knockdown and overexpression efficiency of FBXO44 in selected CRC cell lines. **B.** Western blot analysis was conducted to evaluate the interference efficiency of FBXO44 in the CAC model. **C.** The changes in the relative body weight of BALB/c mice over time are illustrated. **D.** and **E.** Representative images of colons and colon lengths in different treatment groups of mice. **F.** and **G.** Representative images of colons and the number of colonic tumors at least 2 mm or smaller than 2 mm per mouse. **H.** HE staining and IHC analysis of Ki-67, FBXO44, FOXP1, and Cyclin E2 in tumors from different groups. **I.** and **J.** FBXO44 was overexpressed in human colorectal cancer organoids, and organoid number was observed. All data are presented as the means ± SD of three independent experiments. ^ns^*P* > 0.05, **P* < 0.05, ***P* < 0.01, ****P* < 0.001.

**
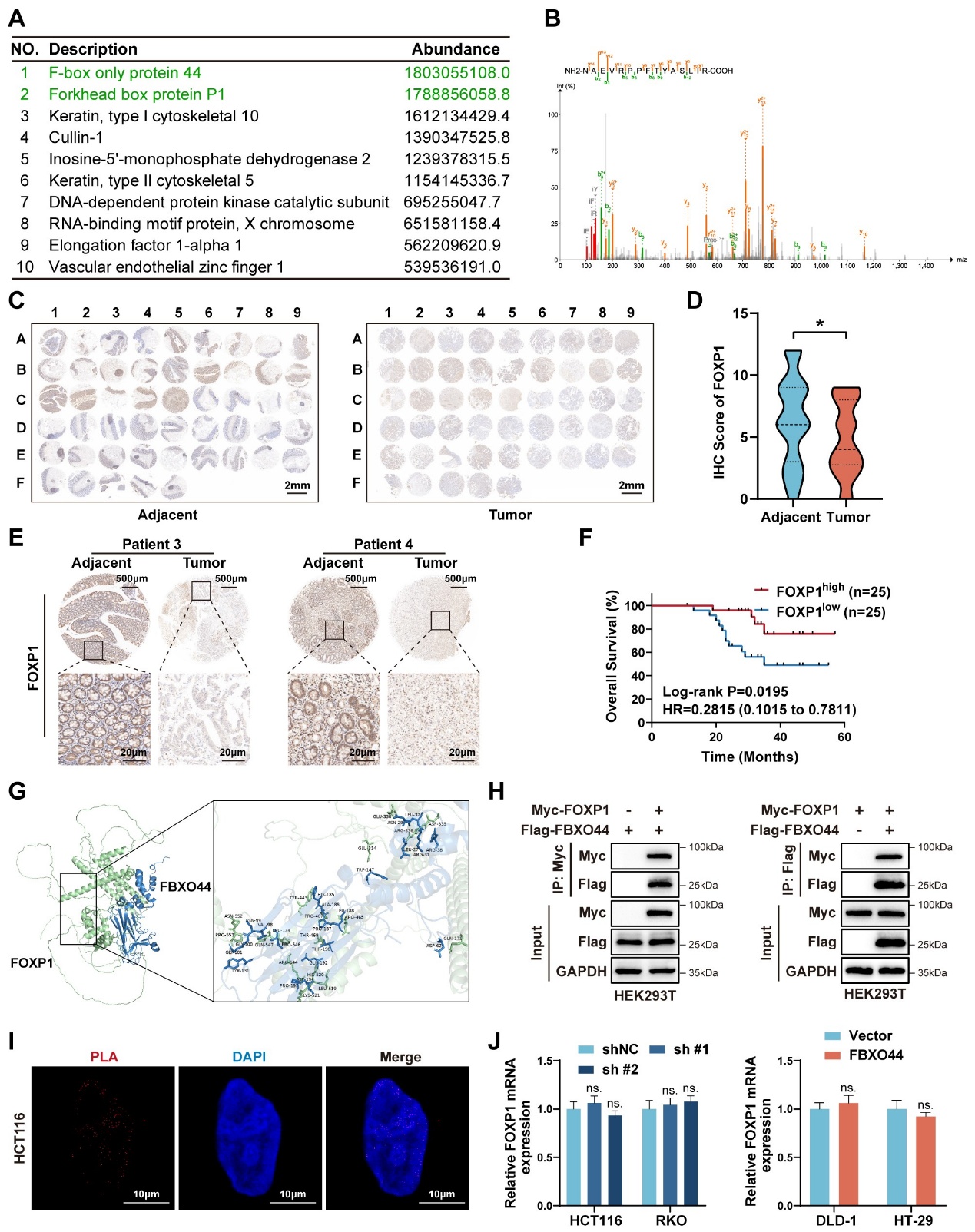
**

**Fig. S2 FBXO44 interacts with FOXP1.**

**A.** List of the top 10 differentially expressed proteins identified by mass spectrometry analysis. **B.** FOXP1 was identified by mass spectrometry analysis. **C.** IHC analysis of FOXP1 expression in CRC TMA. **D.** Violin plot showing IHC scores of FOXP1 in CRC TMA samples. **E.** Representative IHC images showing FOXP1 expression in matched CRC tumor and adjacent normal tissues. **F.** Kaplan–Meier survival analysis comparing patients with low (n=25) and high (n=25) FOXP1 expression based on CRC TMA. **G.** Molecular docking analysis predicted the interaction between FBXO44 and FOXP1. **H.** HEK293T cells were transfected with the indicated plasmids for 24 hours and subsequently treated with MG132 (20 μM) for 6 hours. The interaction between exogenous FBXO44 and FOXP1 was analyzed using co-immunoprecipitation and western blot. **I.** PLA detected the interaction between FBXO44 and FOXP1. **J.** The mRNA expression level of FOXP1 in FBXO44 knockdown and overexpression cells. All data are presented as the means ± SD of three independent experiments. ^ns^*P* > 0.05, **P* < 0.05.

**
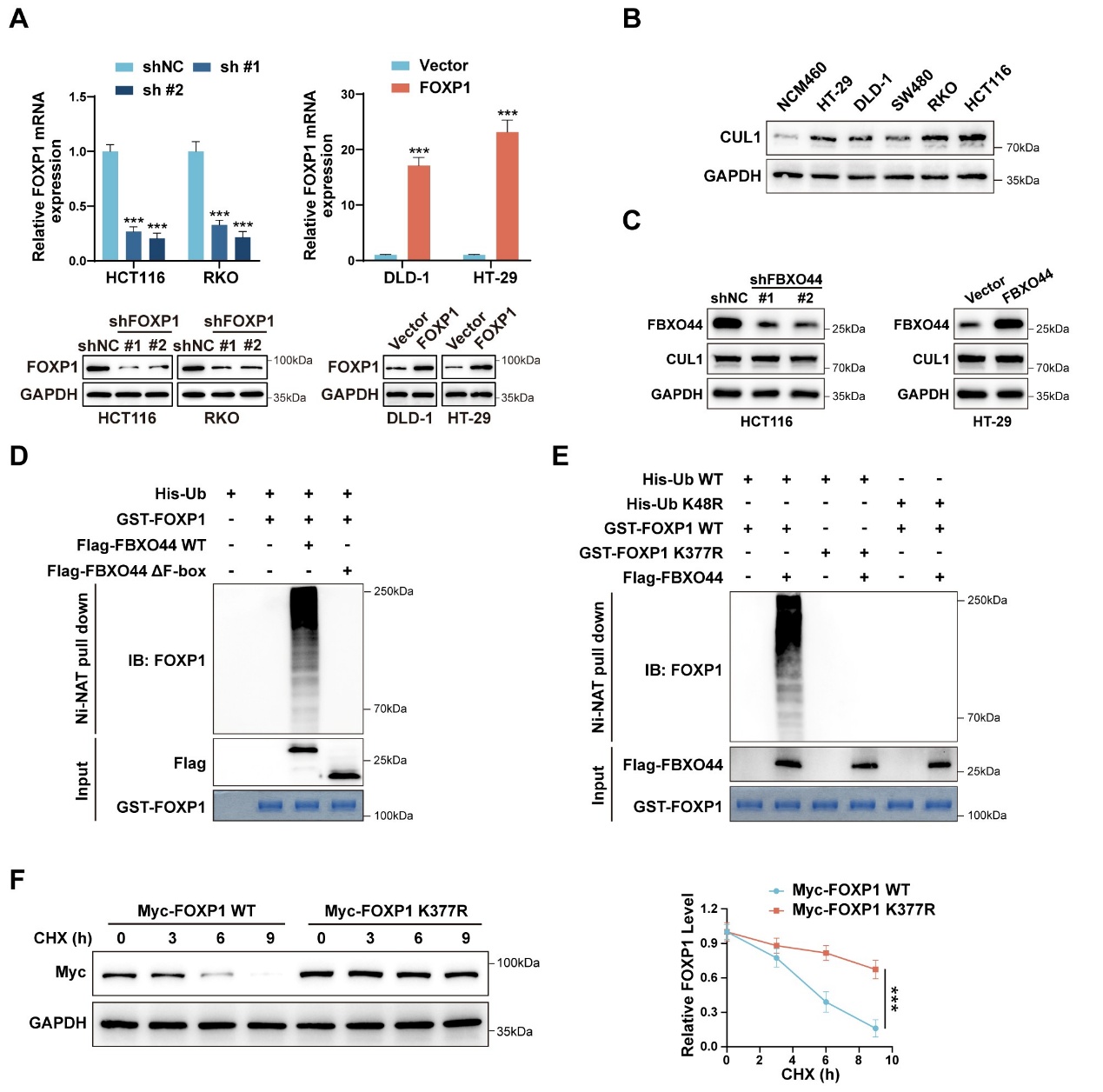
**

**Fig. S3 FBXO44 catalyzes K48-linked ubiquitination of FOXP1 at K377**

**A.** qRT-PCR and western blotting were performed to evaluate FOXP1 knockdown and overexpression efficiency. **B.** Western blot analysis of CUL1 protein levels in the indicated CRC cell lines and NCM460. **C.** CUL1 protein levels were determined in HCT116 cells with FBXO44 knockdown, as well as in HT-29 cells with FBXO44 overexpression. **D.** Purified GST-FOXP1 was incubated with His-tagged ubiquitin and either Flag-FBXO44 WT or Flag-FBXO44 ΔF-box proteins. The reaction mixtures were subjected to Ni-NTA pull down, and ubiquitinated FOXP1 was detected by western blotting using an anti-FOXP1 antibody. **E.** Purified GST-FOXP1 WT or K377R mutant was incubated with Flag-FBXO44 and His-ubiquitin WT or K48R mutant. Reaction mixtures were subjected to Ni-NTA pull down, and ubiquitinated FOXP1 was detected by western blotting using anti-FOXP1 antibody. **F.** HEK-293T cells were transfected with Myc-FOXP1-WT or Myc-FOXP1-K377R plasmid, and then treated with CHX for the indicated durations, cell lysates were subjected to Western blot analysis. Quantification of FOXP1 levels normalized to GAPDH is presented. All data are presented as the means ± SD of three independent experiments. ****P* < 0.001.

**
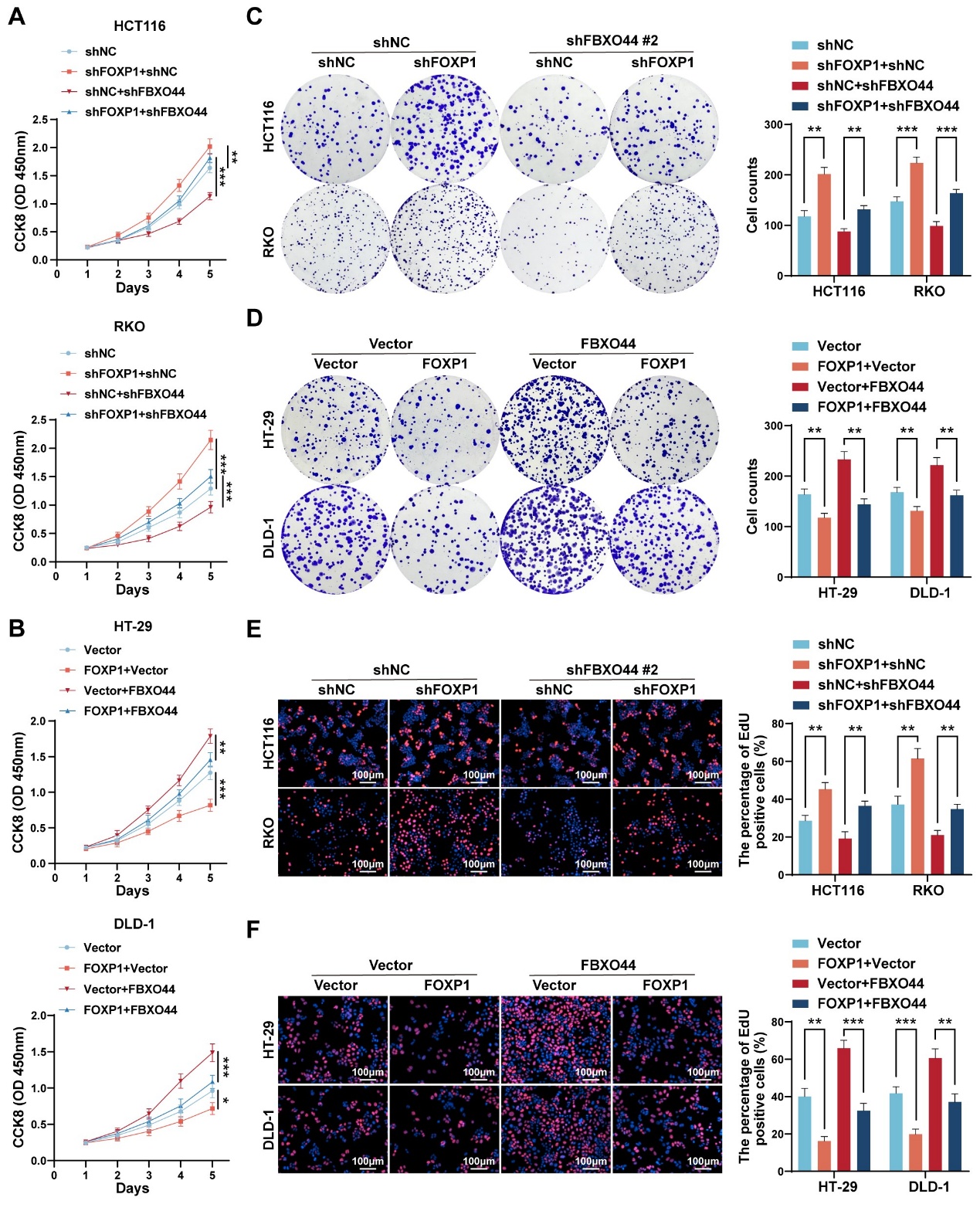
**

**Fig. S4 FBXO44 promotes the proliferation of CRC cells by downregulating FOXP1 *in vitro*. A-F.** CRC cells stably transfected with the indicated lentiviruses were subjected to CCK-8, colony formation, and EdU assays. All data are presented as the means ± SD of three independent experiments. **P* < 0.05, ***P* < 0.01, ****P* < 0.001.

**
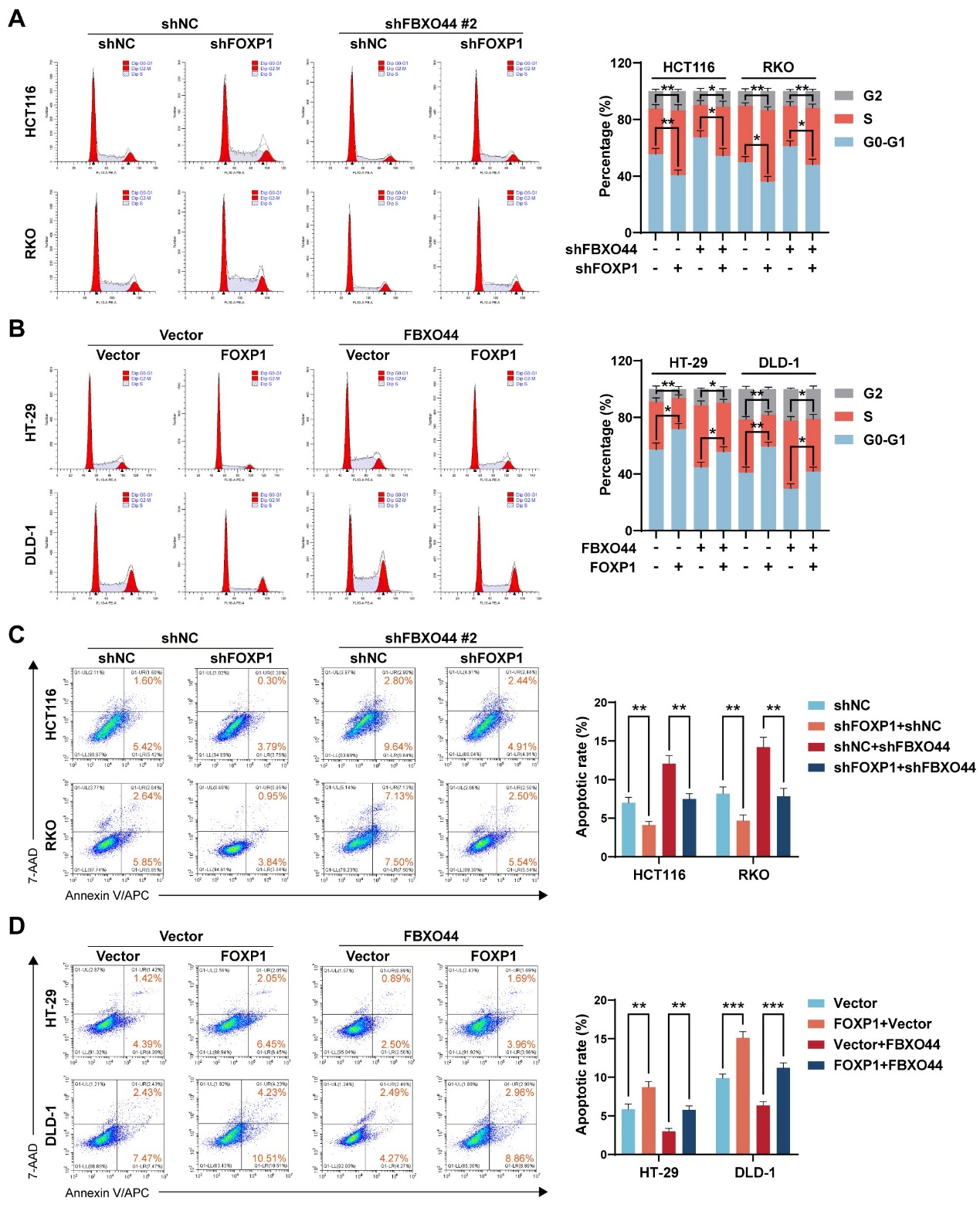
**

**Fig. S5 FBXO44 promotes the proliferation of CRC cells by downregulating FOXP1 *in vitro*.**

**A-D.** CRC cells stably transfected with the indicated lentiviruses were subjected to flow cytometry of cell cycle and apoptosis. All data are presented as the means ± SD of three independent experiments. **P* < 0.05, ***P* < 0.01, ****P* < 0.001.

**
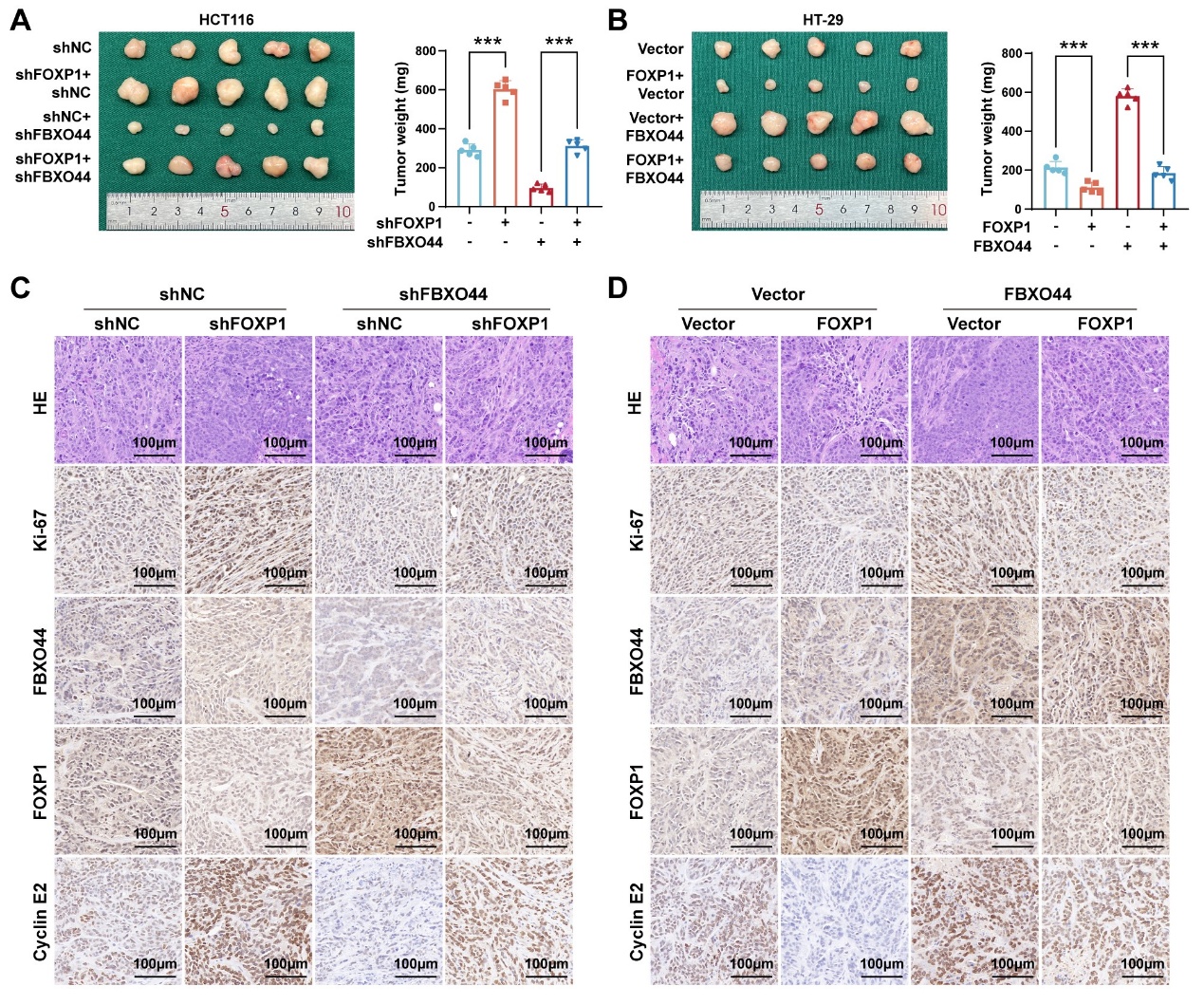
**

**Fig. S6 FBXO44 promotes CRC cell growth by downregulating FOXP1 *in vivo*.**

**A.** and **B.** Representative photographs of subcutaneous xenograft tumors were obtained from nude mice. Average tumor weight was measured at the endpoint. **C.** and **D.** IHC was performed to determine the protein levels of Ki-67, FBXO44, FOXP1, and Cyclin E2 in the xenograft tumors. All data are presented as the means ± SD of three independent experiments. ****P* < 0.001.

**
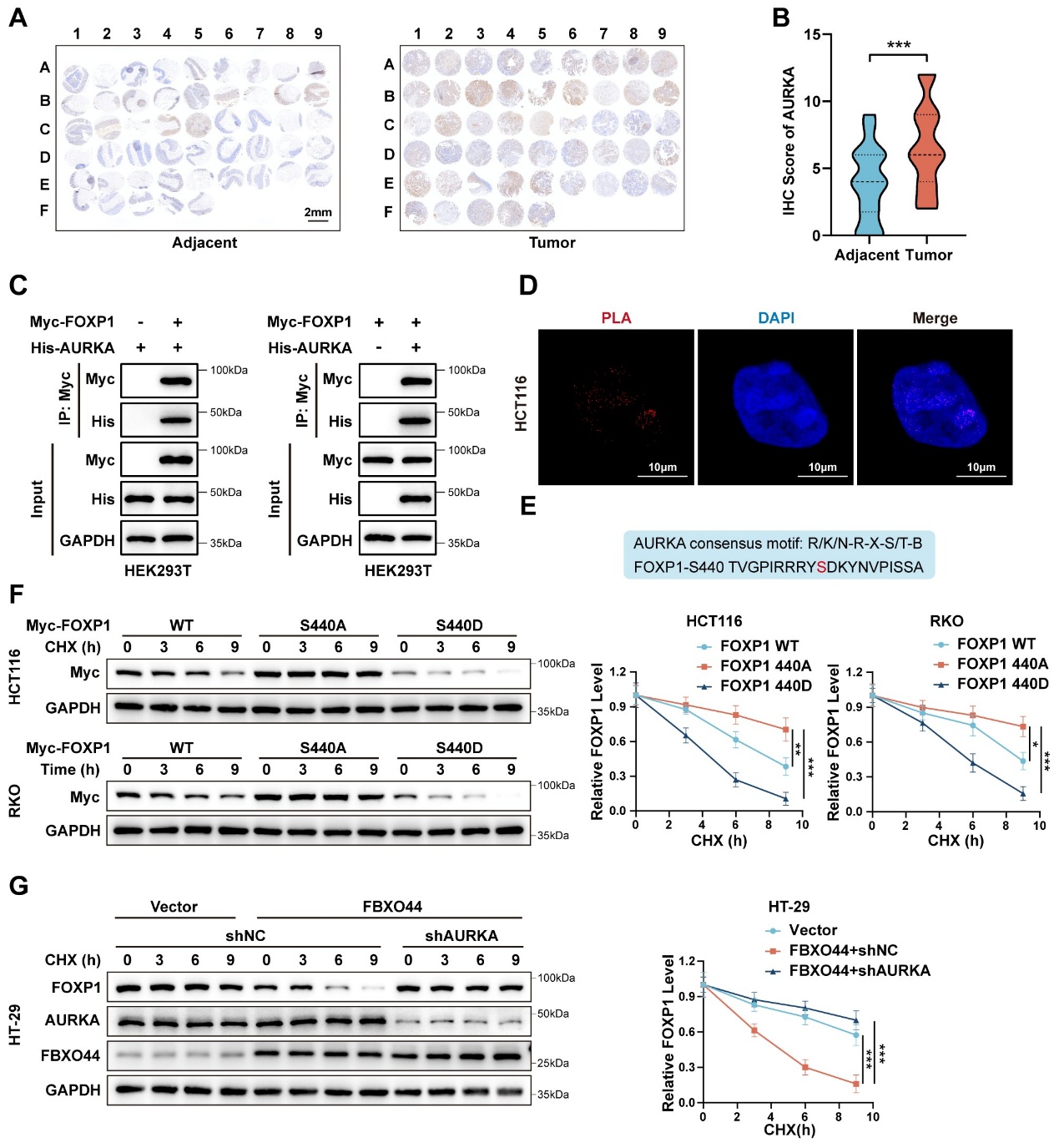
**

**Fig. S7 AURKA-mediated phosphorylation of FOXP1 promotes its ubiquitination and degradation by FBXO44.**

**A.** IHC analysis of AURKA expression in CRC TMA. **B.** Violin plot showing IHC scores of AURKA in CRC TMA samples. **C.** HEK293T cells were transfected with the indicated plasmids for 24 hours and subsequently treated with MG132 (20 μM) for 6 hours. The interaction between exogenous AURKA and FOXP1 was analyzed using co-immunoprecipitation and Western blot. **D.** PLA detected the interaction between AURKA and FOXP1. **E.** The AURKA consensus motif and phosphorylation of FOXP1 at the Ser440 site. **F.** Western blot analysis of FOXP1 expression in HCT116 and RKO cells transfected with Myc-FOXP1 WT, phosphorylation-deficient Myc-FOXP1 S440A or phosphorylation-mimetic Myc-FOXP1 S440D after CHX treatment for the indicated durations. Quantification of FOXP1 levels normalized to GAPDH is presented. **G.** Western blot analysis and quantification of FOXP1 expression in HT-29 cells transfected with vector, shNC, shAURKA or FBXO44 followed by CHX treatment for the indicated durations. All data are presented as the means ± SD of three independent experiments. **P* < 0.05, ***P* < 0.01, ****P* < 0.001.
