# Supplementary material for: FBXO44 Regulates FOXP1 Degradation Through AURKA‐Dependent Phosphorylation to Promote Colorectal Cancer Progression
Source: Adv Sci (Weinh). 2025 Oct 6;12(47):e15458. doi: 10.1002/advs.202415458 (PMC12713037; doi:10.1002/advs.202415458)
Supplement: Supplementary file 2 — Supplemental Table 1 [file ADVS-12-e15458-s003.docx]

| **Table S1 Association of FBXO44 expression with clinicopathological factors (n=80)** | | | | |
| --- | --- | --- | --- | --- |
| **Variable** | **All patients** | **Expression of FBXO44** | | **P value** |
|  |  | **Low** | **High** |  |
| All Cases | 80 | 40 | 40 |  |
| Age (years) |  |  |  |  |
| <60 | 29 | 16 | 13 | 0.4854 |
| ≥60 | 51 | 24 | 27 |  |
| Gender |  |  |  |  |
| Male | 49 | 26 | 23 | 0.4912 |
| Female | 31 | 14 | 17 |  |
| Tumor size (cm) |  |  |  |  |
| <5 | 38 | 24 | 14 | **0.0252** |
| ≥5 | 42 | 16 | 26 |  |
| TNM staging system |  |  |  |  |
| T1 + T2 | 33 | 22 | 11 | **0.0125** |
| T3 + T4 | 47 | 18 | 29 |  |
| Lymph node metastasis |  |  |  |  |
| No | 46 | 19 | 15 | 0.3565 |
| Yes | 34 | 21 | 25 |  |
| Vascular invasion |  |  |  |  |
| No | 60 | 34 | 26 | **0.0389** |
| Yes | 20 | 6 | 14 |  |
| Nerve invasion |  |  |  |  |
| No | 65 | 31 | 36 | 0.3902 |
| Yes | 15 | 9 | 4 |  |
| Distant metastasis |  |  |  |  |
| No | 70 | 36 | 34 | 0.4990 |
| Yes | 10 | 4 | 6 |  |
| CEA (ng/ml) |  |  |  |  |
| <5 | 36 | 21 | 15 | 0.1775 |
| ≥5 | 44 | 19 | 25 |  |

NOTE: CEA carcinoembryonic antigen

P < 0.05 was considered significant. The bold type represents P values smaller than 0.05
