# Supplementary material for: FBXO44 Regulates FOXP1 Degradation Through AURKA‐Dependent Phosphorylation to Promote Colorectal Cancer Progression
Source: Adv Sci (Weinh). 2025 Oct 6;12(47):e15458. doi: 10.1002/advs.202415458 (PMC12713037; doi:10.1002/advs.202415458)
Supplement: Supplementary file 3 — Supplemental Table 2 [file ADVS-12-e15458-s002.docx]

| **Table S2. Antibodies were used in this study.** | | |
| --- | --- | --- |
| **Antigens** | **Manufacturer** | **Application** |
| FBXO44 | Santa Cruz Biotechnology: SC-398020 | 1:100 for WB; 2μg for IP |
| FBXO44 | Sigma-Aldrich: HPA003363 | 1:200 for IF; 1:200 for IHC |
| FOXP1 | Abcam: ab320745 | 1:1000 for WB; 1:1000 for IHC; 1:30 for IP |
| FOXP1 | Novus: NB100-65125 | 1:200 for IF |
| FOXP1 | CST: #4402 | 1:100 for ChIP |
| AURKA | Abcam: ab108353 | 1:2000 for WB; 1:60 for IP; 1:200 for IF |
| AURKA | Proteintech: 10297-1-AP | 1:200 for IHC |
| CUL1 | Proteintech: 12895-1-AP | 1:10000 for WB |
| Ki-67 | Abcam: ab15580 | 1:500 for IHC |
| Cyclin E2 | Abcam: ab40890 | 1:5000 for WB; 1:200 for IHC |
| GAPDH | Proteintech: 60004-1-Ig | 1:50000 for WB |
| Flag | CST: #14793 | 1:1000 for WB; 1:50 for IP |
| Myc | CST: #2276 | 1:1000 for WB; 1:250 for IP |
| His | CST: #12698 | 1:1000 for WB; 1:50 for IP |
| HA | CST: #3724 | 1:1000 for WB |
